# BDNF Augmentation Using Riluzole Reverses Doxorubicin-Induced Decline in Cognitive Function and Neurogenesis

**DOI:** 10.1007/s13311-022-01339-z

**Published:** 2023-01-31

**Authors:** Manal T. Usmani, Robert P. Krattli, Sanad M. El-Khatib, Anh C. D. Le, Sarah M. Smith, Janet E. Baulch, Ding Quan Ng, Munjal M. Acharya, Alexandre Chan

**Affiliations:** 1 Department of Anatomy and Neurobiology, School of Medicine, University of California, Irvine, CA USA; 2 Department of Radiation Oncology, School of Medicine, University of California, Irvine, CA USA; 3 Department of Clinical Pharmacy Practice, School of Pharmacy & Pharmaceutical Sciences, University of California, Irvine, CA USA; 4 Department of Pharmaceutical Sciences, School of Pharmacy and Pharmaceutical Sciences, University of California, Irvine, CA USA

**Keywords:** BDNF, Chemotherapy, Chemobrain, Cognitive function, Riluzole, Neurogenesis

## Abstract

Cancer-related cognitive impairment (CRCI) considerably affects the quality of life of millions of cancer survivors. Brain-derived neurotrophic factor (BDNF) has been shown to promote survival, differentiation, and maintenance of in vivo dentate neurogenesis, and chemotherapy induces a plethora of physiological and cellular alterations, including a decline in neurogenesis and increased neuroinflammation linked with cognitive impairments. In our clinical studies, breast cancer patients treated with doxorubicin (Adriamycin^®^, ADR) experienced a significant reduction in the blood levels of BDNF that was associated with a higher risk of CRCI. Our past rodent studies in CRCI have also shown a significant reduction in dentate neurogenesis accompanied by cognitive impairment. In this study, using a female mouse model of ADR-induced cognitive decline, we tested the impact of riluzole (RZ), an orally active BDNF-enhancing medication that is FDA-approved for amyotrophic lateral sclerosis. ADR-treated mice receiving RZ in the drinking water for 1 month showed significant improvements in hippocampal-dependent learning and memory function (spatial recognition), fear extinction memory consolidation, and reduced anxiety-like behavior. RZ prevented chemotherapy-induced reductions of BDNF levels in the hippocampus. Importantly, RZ mitigated chemotherapy-induced loss of newly born, immature neurons, dentate neurogenesis, and neuroinflammation. In conclusion, this data provides pre-clinical evidence for a translationally feasible approach to enhance the neuroprotective effects of RZ treatment to prevent CRCI.

**Supplementary Information:**

The online version contains supplementary material available at 10.1007/s13311-022-01339-z.

## Introduction

Chemotherapy-related cognitive impairment (CRCI), often referred to as “chemobrain” or “chemofog,” is prevalent in up to 75% of all breast cancer survivors. It encompasses a wide range of symptoms during and after treatment, such as memory loss, inability to concentrate, difficulty in thinking, poor response speed, and executive functioning [1]. These cognitive changes can be subtle but have a detrimental effect on patients’ daily functioning and health-related quality of life [2]. Chemotherapy-induced cognitive decline can pose significant challenges for cancer survivors who wish to resume their day-to-day routine and social roles. Research has suggested that the quality of life and activities of daily living in this group of long-term cancer survivors are immensely affected by subtle neuropsychological changes [3].

Clinical studies have suggested that brain-derived neurotrophic factor (BDNF) is linked with a positive impact on cognition in patients with cancer [3–7]. BDNF exerts a number of neuro-modulatory effects on the CNS and the neurosensory, endocrine, and immune systems, and BDNF is also associated with neuronal repair and survival, dendritic and axonal growth, and long-term potentiation. Numerous studies have reported the possible role of BDNF in the pathogenesis of various cognitive disorders, such as Alzheimer’s disease (AD), where low serum levels have been correlated with AD and mild cognitive impairment, and high serum BDNF levels have been associated with better cognition in healthy older adults [8–11]. In our previous clinical studies, we observed that breast cancer patients [3, 12] receiving doxorubicin and cyclophosphamide reported self-perceived concentration deficits during chemotherapy, and patients experiencing the least reduction of plasma BDNF levels between baseline and end of chemotherapy were most protected from cognitive decline. Adolescent and young adult cancer patients were 3 times more likely to experience cognitive impairment prior to cancer treatment, and their BDNF levels were half of age-matched healthy controls [13]. As chemotherapy can be neurotoxic, it is postulated that the effects of chemotherapy on BDNF expression can persist long after the completion of chemotherapy and in cancer survivors, resulting in persistent cognitive decline post-chemotherapy [14]. Loss of BDNF may also accelerate aging, which is postulated to be a mechanism that leads to cognitive decline in patients receiving chemotherapy [15].

Supplementation of BDNF has been shown to protect synapses against various toxic insults in neurodegenerative diseases, including AD, Huntington’s disease, ALS, and Parkinson’s disease [16] in animal models. These existing data provide strong justification for the need to study the viability of augmenting BDNF levels to reverse chemobrain. To study the viability of this strategy, we have conducted a pre-clinical study to evaluate the efficacy of riluzole (RZ), an orally bioavailable agent that has been shown to increase BDNF [17] and improve cognitive function in AD models [18]. We hypothesize that RZ treatment will reduce the onset and severity of the chemotherapy-induced cognitive decline. We also hypothesize that RZ will augment BDNF levels and that BDNF plays a major contributing factor in protecting against adverse impacts on neurogenesis, microglial activation, and ultimately cognition.

## Materials and Methods

Details on materials, experimental methods, behavior, and immunostaining protocols are provided in the Supplemental Information section.

### Animals and Treatments

**Fig. 1:**
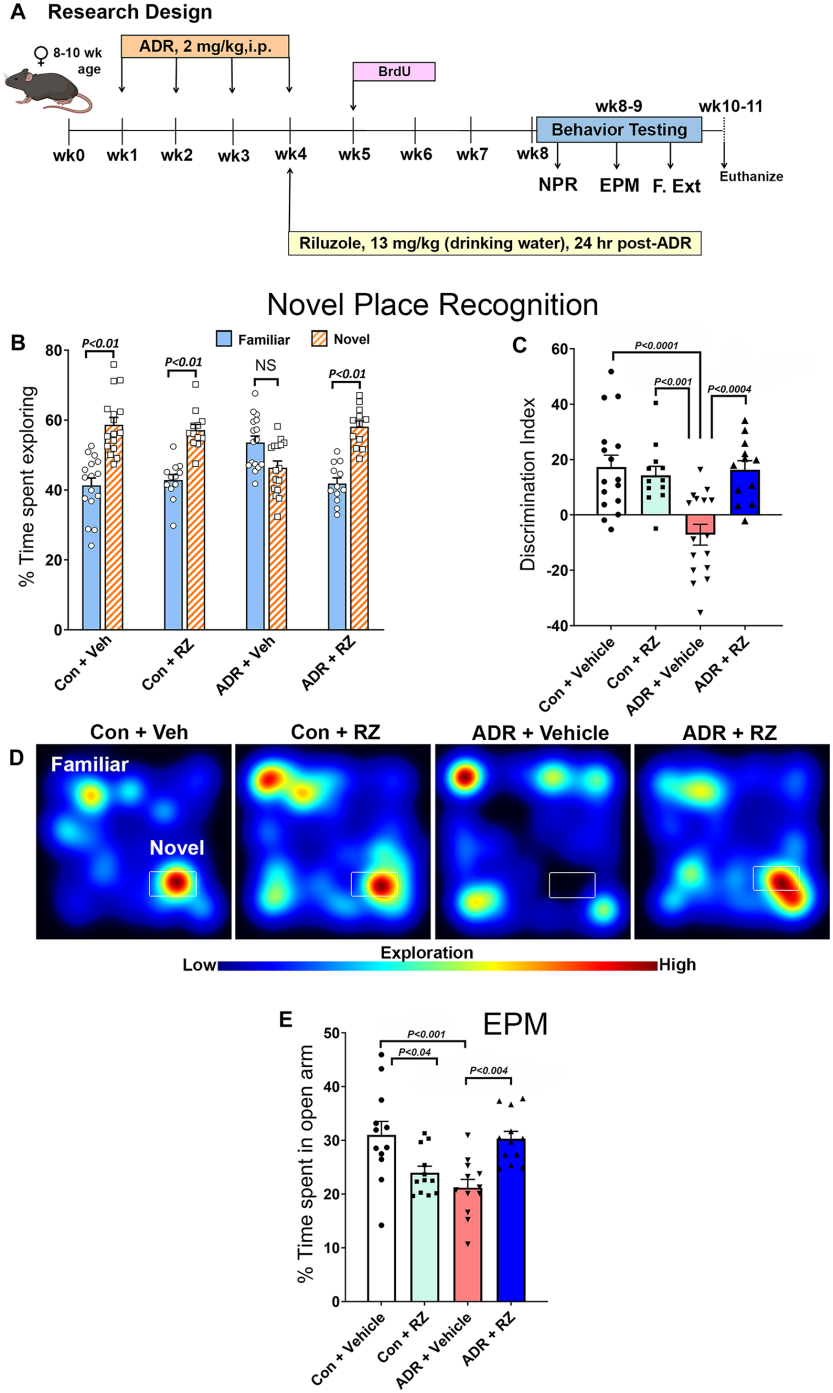
Treatment with riluzole reverses chronic chemotherapy-induced cognitive dysfunction. **A** Schematic representation of research design: Eight to ten-week-old wild-type (C57BL/6 J) female mice were injected with vehicle (vehicle, saline) or an anthracycline, Adriamycin (ADR, doxorubicin hydrochloride, 2 mg/kg, i.p.), once weekly for 4 weeks. Twenty-four hours after the last ADR injection, mice were given riluzole (RZ) treatment in the drinking water (13 mg/kg) and continued on RZ till the end of the study. ADR-treated animals that received drinking water served as the vehicle group. To assess the in vivo neurogenesis, 1 week after the last ADR injection, mice were administered with BrdU (5-Bromo-2′-deoxyuridine, 50 mg/kg, i.p., once daily for 6 days). One month after initiation of RZ treatment, mice were administered a spatial memory retention test (novel place recognition, NPR), an anxiety test (elevated plus maze, EPM) followed by a fear extinction memory consolidation task (F. Ext.). After the completion of cognitive testing, mice were euthanized, and brains were collected for the biochemical and immunohistochemistry analyses. **B** ADR-treated mice spent significantly less time exploring novel spatial location. Time spent exploring the novel placements of objects during the test phase of the NPR task show that control + vehicle, control + RZ, and ADR + RZ mice spent significantly more time exploring novel versus familiar location whereas ADR + vehicle mice spent comparable time exploring both locations indicating an inability to recognize the novel location. **C** The tendency to explore a novel spatial location was derived from the discrimination index, calculated as ((novel location exploration time/total exploration time) – (familiar location exploration time/total exploration time)) × 100. ADR treatment significantly impaired cognitive function as shown by the reduced preference toward the novel place in the ADR + vehicle mice compared to the control + vehicle, control + RZ, and ADR + RZ mice. Importantly, ADR-exposed mice receiving RZ treatment did not show a decline in spatial exploration. **D** Examples of heat maps depicting animals exploring novel or familiar spatial locations in each experimental group during the NPR task. **E** ADR + vehicle mice showed increased anxiety levels as indicated by reduced time (percentage) spent in the open arms on the EPM tasks. In contrast, control + vehicle and ADR + RZ-treated mice showed significantly higher time spent in the open arms. Data are presented as mean ± SEM (*N* = 12–16 mice per group). *P* values were derived from a two-way ANOVA and Bonferroni’s multiple comparisons test

All animal experiments were approved by the Institutional Animal Care and Use Committee (IACUC) and according to the NIH guidelines. Our past clinical studies have shown a positive correlation between the low BDNF levels and the severity of CRCI in female breast cancer patients [3, 12]. Thus, for this pre-clinical study, we used adult female mice. Four-month-old female wild-type mice (C57BL/6 J) were purchased from Jackson Laboratories. Mice were group housed (4 mice per cage), kept on a standard light–dark cycle (12 h each) at 20 °C ± 1 room temperature, and 70% ± 10 humidity and given a standard rodent chow diet (Envigo Teklad 2020X) by the University Laboratory Animal Recourses (ULAR). Mice received Adriamycin injections (ADR, doxorubicin hydrochloride, Sigma) freshly made in saline (2 mg/kg, once weekly, i.p.) for 4 weeks as shown in the research design (Fig. 1A). Riluzole (2-amino6-(trifluoromethoxy)benzothiazole, Selleckchem) was dissolved in filter sterilized (0.2 µm, Millipore), warm RO water (reverse osmosis, ULAR) with constant stirring (1 to 2 h at 45 °C) at a stock concentration of 600 µg per ml. The stock solution was kept frozen at − 20 °C until use. The working solution (60 µg per ml) of RZ was prepared twice a week by diluting the stock with sterile RO water. Mice had ad libitum access to either vehicle (sterile RO water) or RZ solution (13 mg/kg per mouse, per day as described [18–20]) throughout the duration of the study. Mice received RZ treatment 24 h after the last ADR injection (Fig. 1A). To evaluate the impact of ADR or RZ treatment on in vivo neurogenesis, 1 week after the last ADR injection, mice were administered BrdU (5-bromo-2′-deoxyuridine, 50 mg/kg, i.p., Sigma) made in phosphate-buffered saline (PBS, pH 7.6, Sigma) once daily for 6 days. Mice were divided into four experimental groups (*N* = 12 to 24 mice per group) including saline-treated control mice receiving the RO water (Con + Veh), ADR-treated mice receiving the RO water (ADR + Veh), saline-treated control mice receiving RZ (Con + RZ), and ADR-treated mice receiving RZ (ADR + RZ).

### Cognitive Testing

To determine the effect of RZ-mediated BDNF augmentation on cognitive function after chronic chemotherapy, mice were administered cognitive function and anxiety-like behavior tests 4 weeks after the initiation of RZ treatment. The details about test protocols are provided in the Supplemental Information section. Over 2 to 3 weeks, cognitive function tasks included novel place recognition (NPR), elevated plus maze (EPM) for anxiety-like behavior, and, lastly, fear extinction memory consolidation task (FE). The NPR task is dependent on the intact hippocampal function and assesses episodic and spatial memory function [21–23]. NPR determines the ability of animals to explore novel placement of the objects in an unrestricted, non-invasive open environment [22, 23] that is calculated as discrimination index (DI = (novel/total exploration time) − (familiar/total exploration time)] × 100). A positive DI indicates that animals spent more time exploring novel spatial locations. In contrast, a negative or zero index shows that animals exhibited little or no preference for the novel placements and spent equal time exploring familiar and novel places. Data for the open field activity (Suppl. Fig. S1) on the first day of NPR task (habituation phase), and total time spent exploring both placements of the objects (Suppl. Fig. S2) were also derived from the NPR task. EPM measures anxiety levels based on animal exploration in the elevated open arms versus closed (dark) arms under a brightly lit environment. Anxious animals will spend more time in closed arms compared with open arms [24].

After completing NPR and EPM tasks and about 72 h of break, animals were administered the FE task. This task determines if chronic chemotherapy or RZ treatment affects hippocampal-dependent fear conditioning and memory consolidation process, an active process of dissociating learned responses to prior adverse events [25–27]. Briefly, on the first day of the conditioning phase, mice were presented with three pairs (evenly spaced) of auditory stimulus co-terminating with a mild foot shock. Twenty-four hours later, on the subsequent 3 days (extinction training phase), animals were presented with 20 tones while in the same contextual environment (odor and visual cues). Twenty-four hours later, the fear test was administered on the final day, where animals were presented with only three tones in the same context. Animals’ freezing behavior was recorded using a ceiling-mounted camera in the FE test chamber and recorded by an automated freezing measurement module (FreezeFrame, Coulbourn Instruments). The percentage of time each mouse spent freezing during the tone was then calculated for conditioning, extinction training (average of five tones, four data points per day), and testing phases. Thus, a combination of these cognitive function tasks provides us with rigorous tools to determine the impact of chemotherapy and RZ. Protocol details are provided in the Supplemental Information section.

### Immunohistochemistry, Confocal Microscopy, and In Silico Volumetric Quantification

After the completion of cognitive function tests, mice were euthanized (intra-cardiac perfusion) using saline with heparin (10 U/ml, Sigma) and 4% PFA made in 100 mM PBS, pH 7.4 (paraformaldehyde, Sigma; phosphate-buffered saline, Gibco). Brains were fixed overnight at 4 °C in 4% PFA. Tissues were then cryo-protected (10 to 30% sucrose made in 100 mM PBS, pH 7.4 and 0.02% sodium azide, Sigma) and cryo-sectioned using a cryo-stat (HN525 NX, Microm, Epredia, Germany) at the thickness of 30 µm (coronal). To determine the impact of chronic chemotherapy and RZ treatments on the function of the neurogenic niche, serial coronal brain sections (2 to 3 sections per brain, 8 to 10 brains per group) through the hippocampal formation were stained using free-floating immunofluorescence protocols as described [28]. Doublecortin (DCX) staining was performed to label newly born, immature neurons [29] using a rabbit anti-DCX primary (1:200; Abcam) and a donkey anti-Rabbit Alexa Fluor 568 secondary antibodies. DCX-positive cells were visualized using fluorescence microscopy as red. For the BrdU-NeuN dual-immunofluorescence staining, tissues were permeabilized to recover the BrdU antigen and stained using rat anti-BrdU (1:150; Abcam) and rabbit anti-NeuN (1:500, Millipore) primary antibodies. The fluorescence color was developed using donkey anti-rat Alexa Fluor 488 (1:150, Invitrogen) and donkey anti-rabbit Alexa Fluor 568 (1:500, Invitrogen) secondary antibodies. BrdU-positive cells were visualized using fluorescence microscopy as green and NeuN-positive cells as red. Activated microglia were labeled by IBA1-CD68 dual immunofluorescence staining (pan microglial marker ionized calcium-binding adapter molecule 1, IBA1; and lysosomal protein in the activated microglia, CD68). Briefly, coronal tissues were permeabilized using 0.3% Tween-20 (Sigma) in PBS followed by 3% hydrogen peroxide (Sigma) and 10% methanol (Sigma) treatments. Tissues were then blocked using 4% bovine serum albumin (Jackson ImmunoResearch) and 0.3% Tween-20 in PBS followed by primary antibody incubation overnight (rabbit anti-IBA1, 1:500, Wako; and rat anti-mouse CD68, 1:500, AbD Serotec, Bio-Rad). The fluorescence color development was facilitated by goat anti-rat Alexa Fluor 647 and goat anti-rabbit Alexa Fluor 488 (1:1000 each dilution, Abcam) secondary antibodies. IBA1-positive cells were visualized using fluorescence microscopy as green and CD68-positive puncta cells as magenta. A detailed protocol is provided in the Supplemental Information section.

Single- (DCX) or dual-(BrdU-NeuN; IBA1-CD68) immunofluorescent sections were imaged using a laser-scanning confocal microscope (Nikon Eclipse Ti C2) equipped with a 40 × oil-immersion objective lens (1.0 NA) and NIS element AR module (v4.3, Nikon). The high-resolution (1024 to 2048 p) z stacks (1 µm thick) were acquired through the 25–30 µm thick brain sections. Unbiased deconvolution for the fluorescent z stacks and in silico volumetric quantification were carried out as described previously [30–33] and are provided in the Supplemental Information section. An adaptive, 3D blinded deconvolution method (ClearView, Imaris v9.2, BitPlane, Inc.) was used to deconvolute and facilitate the fluorescence signal resolution with respective fluorescent wavelengths (510 nm, green; 594 nm, red; 447 nm, UV; and 647 nm for IR ranges). The deconvoluted IMS format images were analyzed using a 3D algorithm-based Imaris module (v9.2). In Imaris, BrdU, NeuN, IBA1, and CD68 were 3D modeled using the surface-rendering tool to create the volume of individual glial or neuronal cells. Using an unbiased, dedicated co-localization channel, the number of BrdU^+^ cells co-labeled with NeuN^+^ neurons was individually enumerated. Similarly, IBA1 and CD68 co-localization was determined for the activated microglia. The number of DCX^+^ cells was quantified using the spot analysis tool. All Imaris-based in silico analyses were conducted using automated batch processing modules. The criteria were applied uniformly for all experimental groups by an experimenter blinded to the group IDs to avoid bias.

### BDNF ELISA

To determine the impact of RZ on the hippocampal BDNF, mice receiving ADR or RZ were euthanized for 4 weeks after the initiation of RZ treatment, and BDNF ELISA was performed as described [34]. Brains were immediately extracted from the skull (*N* = 8 to 9 mice per group). The hippocampus was microdissected from each cerebral hemisphere, flash-frozen by immersing the cryo-vials in the liquid nitrogen, and stored at − 80 °C until assayed. Each hippocampus was weighed and transferred into 500 μl ice-cold lysis buffer (NPER, Neuronal Protein Extraction Reagent, ThermoScientific) containing sodium orthovanadate (0.5 mM, Santa Cruz), phenyl-methylsulfonyl fluoride (PMSF, 1 mM, Santa Cruz), aprotinin (10 μg/ml, Santa Cruz), and leupeptin (1 μg/ml; Santa Cruz). Tissues were then sonicated individually and centrifuged at 4 °C, and the supernatants were collected and diluted at 1:5 or 1:10 with ice-cold Dulbecco’s PBS (Gibco). The supernatants were acidified to pH 2.6 and then neutralized to pH 7.6. The BDNF levels were assayed using a commercially available ELISA kit (E-EL-M0203, Elabscience Biotechnology) and uncoated ELISA plates (Nunc MaxiSorp, Biolegend). The colorimetric measurements were performed at 450 nm wavelength using a microplate reader (BioTek SynergyMx).

### Statistical Analysis

All data are presented as the mean ± SEM. The statistical analyses of cognitive function, biochemical, and immunohistochemical data were conducted using a two-way ANOVA (GraphPad Prism, v8.0). For the chemotherapy or RZ treatment analysis, a two-way ANOVA and Bonferroni’s multiple comparisons tests were performed as standard for the cognitive tests [24, 35, 36]. The Wilcoxon matched-pairs signed-rank test was used to compare the exploration of familiar versus novel places by the same animals in the NPR task without normality assumptions. Pearson’s correlation coefficients were calculated to assess the relationship between BDNF levels and cognitive function. A three-way ANOVA and Bonferroni’s multiple comparisons tests were performed to analyze the fear extinction test data. All statistical analyses were considered significant for a value of *P* ≤ 0.05. 

## Results

### Riluzole Treatment Mitigates Chemotherapy-Induced Cognitive Impairments and Anxiety-Like Behavior

Adult WT female mice received chronic ADR treatment (once weekly for 4 weeks) followed by RZ in drinking water (Fig. 1A). One month after RZ treatment, mice were handled, habituated, and tested on the hippocampal-dependent NPR task (Fig. 1B, C). The open field activity of animals in the open arena was monitored for the day 1 of the habituation phase (no objects). We did not find significant differences between the experimental groups for the total distance traveled and time spent in the central zone (60% of central area) for the open field activity (Suppl. Fig. S1) indicating the absence of neophobic behavior during cognitive testing. During the test phase, the total time exploring both familiar and novel placements of objects did not differ significantly between the experimental groups (Suppl. Fig. S2). Comparison of the animal exploration of the familiar and novel spatial location times (Fig. 1B) revealed significant differences between control + vehicle, control + RZ, and ADR + RZ mice (*P* < 0.01), but not for the ADR + vehicle mice, indicating no differences in an animal’s preference for the novel location. The level of preference for novelty was then calculated as the discrimination index (DI; see “Materials and Methods”) (Fig. 1C). The two-way ANOVA found a significant interaction between RZ and ADR treatments (*F*_(1, 52)_ = 11.87, *P* = 0.001) as well as an RZ effect (*F*_(1, 52)_ = 7.07, *P* = 0.01) and ADR effect (*F*_(1, 52)_ = 8.57, *P* = 0.005). Furthermore, ADR-treated mice receiving vehicle (ADR + vehicle) showed a significantly reduced preference to explore the novel location compared to the control + vehicle (*P* < 0.0001), control + RZ (*P* < 0.001), and ADR + RZ (*P* < 0.0004) mice. This behavior is also reflected in the heat map of mouse exploration activity for the novel or familiar spatial locations (Fig. 1D). Importantly, ADR-treated mice receiving RZ did not show reductions in the discrimination index, and exploration for the novel location was comparable to the control + vehicle and control + RZ mice (*P* ≥ 0.99, Fig. 1C).

To determine whether chronic ADR and RZ treatments affected anxiety-like behavior, all groups of female mice were administered an elevated plus maze (EPM) task (Fig. 1E). The EPM task evaluates the preference for exploring either open or the closed (dark) arms of the maze under the bright ceiling lights. Anxious mice would show a higher preference for the closed versus open arms to avert brightly lit space. Using the two-way ANOVA, we observed a significant interaction between RZ and ADR treatments (*F*_(1, 44)_ = 21.7, *P* < 0.0001), though we neither observed a significant RZ effect (*F*_(1, 44)_ = 0.35, *P* = 0.56) nor ADR effect (*F*_(1, 44)_ = 1.03, *P* = 0.32). ADR + vehicle-treated mice spent significantly less time in the open arms than the control + vehicle group (*P* < 0.001). Control + RZ-treated mice also showed reduced time spent in the open arms compared to the control + vehicle group (*P* < 0.04). In contrast, ADR + RZ-treated mice showed significantly increased time spent in the open arms (*P* < 0.004, Fig. 1E) than ADR + vehicle groups indicating reduced anxiety-like behavior following RZ treatment.

**Fig. 2:**
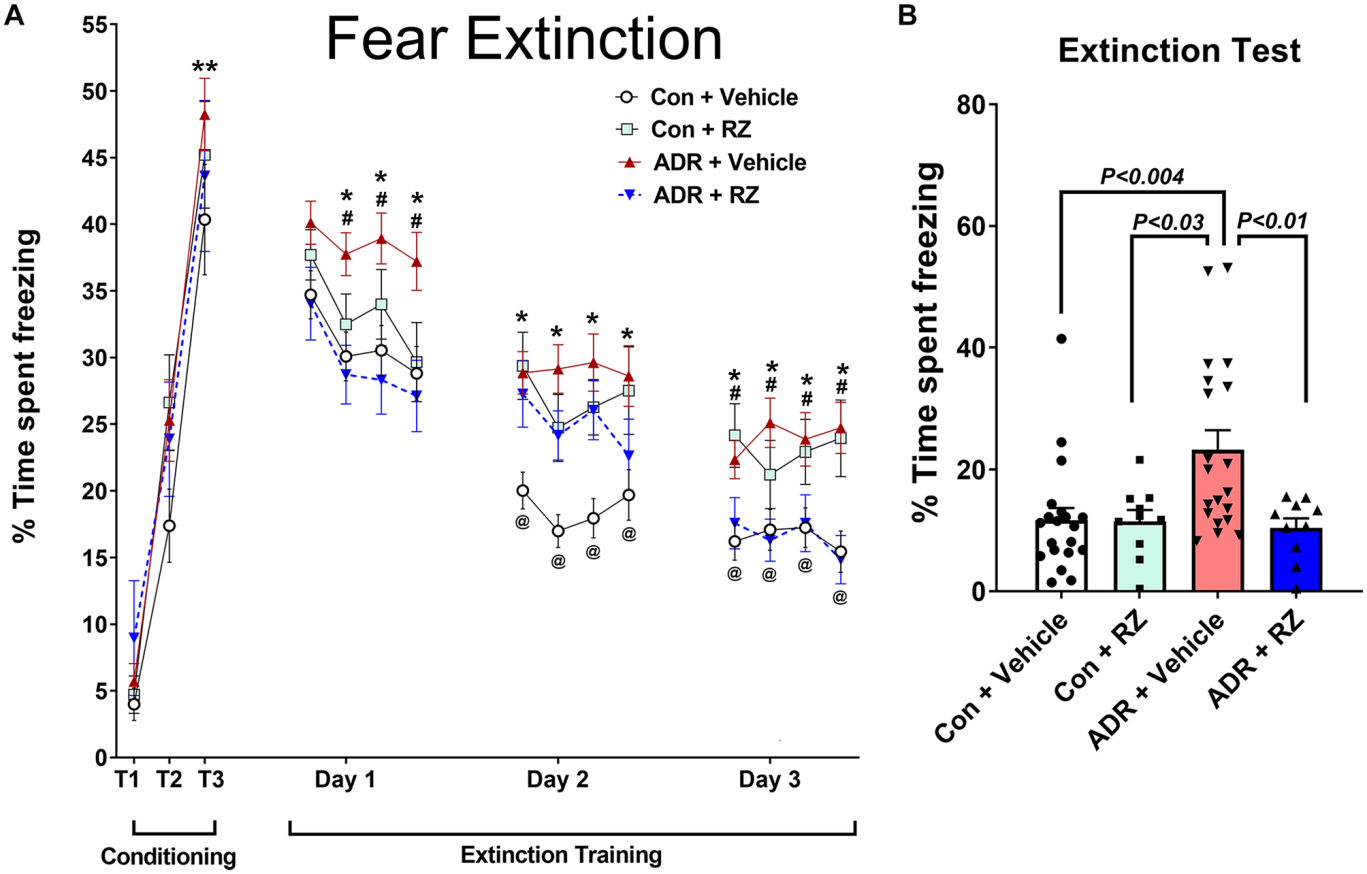
Riluzole treatment alleviates chemotherapy-induced deficits in fear memory consolidation. **A** Treatment with either ADR or RZ did not impair the acquisition of conditioned fear memory as shown by the elevated freezing following a series of three-tone and shock pairings (80 dB, 0.6 mA, T1–T3). Subsequently, 24 h later, fear extinction training was administered every 24 h (20 tones) for 3 days. Each data point for the days 1–3 are presented as average of percentage time freezing for 5 tones (4 data points per day). All mice showed a gradual decrease in freezing behavior (days 1–3); however, ADR + vehicle mice spent a significantly higher time freezing compared with control + vehicle or ADR + RZ mice. For days 2 and 3, control + RZ mice also showed increased freezing compared to Con + Vehicle group. **B** Twenty-four hours after the extinction training, on the extinction test, control + vehicle and control + RZ mice showed abolished fear memory (reduced freezing) compared with ADR + vehicle mice. Importantly, ADR-treated mice receiving RZ (ADR + RZ) were able to successfully abolish fear memory (reduced freezing) compared with the ADR + vehicle group. Data are presented as mean ± SEM (*N* = 10–20 mice per group). *P* values were derived from a three-way ANOVA and Bonferroni’s multiple comparisons test. *P < 0.01, ADR + vehicle vs. control + vehicle; ^#^*P* < 0.01 ADR + vehicle vs. ADR + RZ. For day 2, ^@^*P* < 0.02, control + RZ vs. Control + vehicle. For day 3, ^@^*P* < 0.02, control + RZ vs. Con + vehicle; and control + RZ vs. ADR + RZ groups. **P < 0.0001, T1 vs T3 for all experimental groups

In the past, we reported chemotherapy-induced impairments in contextual fear memory and fear extinction memory consolidation [29, 37]. Thus, to evaluate if RZ treatment exerts beneficial effects or not, animals were administered the fear extinction memory consolidation task (FE, Fig. 2). During the conditioning phase of FE testing, all groups of female mice (control + vehicle, control + RZ, ADR + vehicle, and ADR + RZ) showed comparable associative learning as indicated by increased time spent freezing during the tone-shock conditioning phase (Fig. 2A; conditioning day tone-shock pairings, *T*_1_, *T*_2_, and *T*_3_; 40 to 48% freezing on *T*_3_). The three-way ANOVA found a significant impact of tone-shock pairings on the freezing behavior (*F*_(2, 168)_ = 107.2, *P* < 0.0001). The comparison of freezing between the *T*_1_ and *T*_3_ tone-shock pairings showed a significant increase for each treatment group (*P* < 0.0001) indicating the impact of conditioning. Twenty-four hours later, during the extinction training phase (days 1–3, Fig. 2A), mice were presented with 20 tones per day (5 s intervals) in the same context as the conditioning phase while no foot shock was administered. Each data point is presented as an average of five tones (four data points for 20 tones per day, Fig. 2A). For each extinction training day, we conducted a three-way ANOVA to determine the impact of ADR, RZ, or time on the extinction training and re-learning process. We did not find a significant triple interaction effect (time × RZ × ADR) for freezing behavior over the extinction training phase (days 1–3; *P* ≥ 0.78). We found significant RZ × ADR interaction for each of the extinction training days (*P* < 0.0001). For the fourth data point in the extinction training day for each treatment group, we found a significant time effect (*F*_(2, 883)_ = 16.23, *P* < 0.0001) and RZ effect (*F*_(1, 883)_ = 5.33, *P* = 0.021) but no ADR effect (*F*_(1, 883)_ = 1.98, *P* = 0.16). Multiple comparisons between animal groups for the day 1 extinction training showed significantly higher freezing in the ADR + vehicle groups compared to control + vehicle (*P* < 0.01) and ADR + RZ (*P* < 0.01) groups. For the day 2 training, the ADR + vehicle group continued elevated freezing behavior compared to the control + vehicle group (*P* < 0.01). Interestingly, for day 2, the control + RZ and ADR + RZ groups also showed elevated freezing compared to the control + vehicle group (*P* < 0.02). Subsequently, for extinction training day 3, the ADR + vehicle group showed significant freezing compared to the control + vehicle and ADR + vehicle groups (*P* < 0.01). The control + RZ group also showed increased freezing compared to the control + vehicle group (*P* < 0.02). Importantly, RZ treatment to the chronically exposed ADR animals mitigated impairments in the ability to dissociate the learned response (freezing) to a prior aversive event (tone-shock pairing).

During the 24-h post-extinction training phase, the female mice were administered extinction testing in the same testing environment used for extinction training (Fig. 2B). The two-way ANOVA found a significant interaction between ADR and RZ treatments (*F*_(1, 56)_ = 5.12, *P* = 0.027) as well as RZ effect (*F*_(1, 56)_ = 5.41, *P* = 0.024) but no ADR effect (*F*_(1, 56)_ = 3.5, *P* = 0.07). Moreover, multiple comparisons showed significant differences between the control + vehicle and ADR + vehicle groups (*P* = 0.004); control + RZ and ADR + vehicle groups (*P* = 0.03); and ADR + RZ and ADR + vehicle groups ( *P* = 0.01). These data indicate that ADR + vehicle mice failed to abolish fear memories during this retrieval testing and exhibited increased freezing that was ameliorated by the RZ treatment (Fig. 2B).

These cognitive function and anxiety-like behavior data demonstrated that chronic chemotherapy-induced impairments were mitigated by RZ treatment.

### Riluzole Restores In Vivo BDNF Levels in the ADR-Treated Mice Brains

**Fig. 3:**
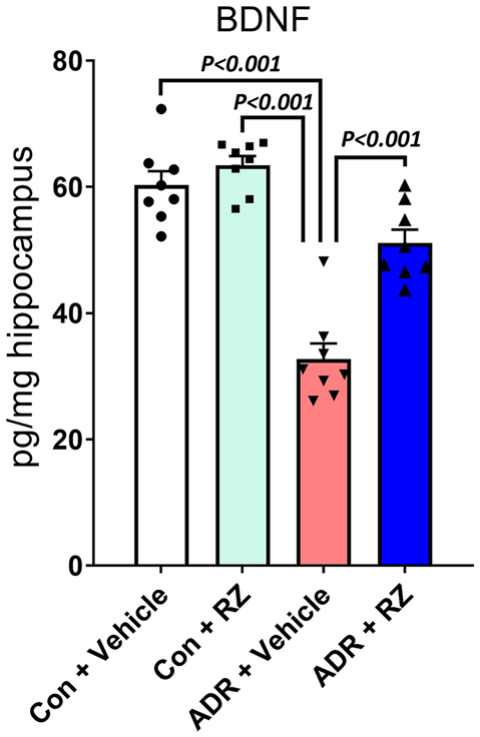
Riluzole treatment restores hippocampal BDNF in the chemotherapy-exposed mice. Eight to ten-weeks-old WT female mice received chronic ADR treatment (2 mg/kg, i. p., once weekly for 4 weeks) followed by riluzole (RZ) treatment (13 mg/kg) in drinking water for 4 weeks. An ELISA-based estimation of BDNF from micro-dissected mice hippocampus showed chemotherapy-induced reductions in the ADR + vehicle group. Importantly, RZ treatment to the ADR-exposed mice showed significant restoration of BDNF levels. Data are presented as mean ± SEM (*N* = 8 mice per group). *P* values were derived from a two-way ANOVA and Bonferroni’s multiple comparisons test

Our past human studies have shown that the reduction of BDNF levels is linked with acute and persistent self-reported cognitive impairments in cancer survivors treated with cytotoxic agents including cyclophosphamide and ADR [3, 12]. In the mouse model of AD, RZ treatment has been shown to improve cognitive function [20]. To link favorable cognitive and behavioral outcomes in the ADR-treated female mice receiving RZ, we conducted an ELISA-based estimation of BDNF in the micro-dissected hippocampus (Fig. 3). Using a two-way ANOVA, we found a significant an RZ × ADR interaction (*F*_(1, 28)_ = 13.36, *P* = 0.001), an RZ effect (*F*_(1, 28)_ = 26.61, *P* < 0.0001), and an ADR effect (*F*_(1, 28)_ = 90.97, *P* < 0.0001). Chronic ADR treatment led to an approximate 45% decrease in the BDNF levels compared with either control + vehicle or control + RZ groups (*P* < 0.001). RZ treatment significantly restored BDNF levels in the mice exposed to chronic ADR (*P* < 0.001). The correlation analysis did not reveal a relationship between BDNF levels and the percentage of time spent on the open arm (EPM) for the control + RZ group (*R* =  − 0.047). In contrast, a positive correlation was found between the BDNF levels and the percentage of time spent on the open arms for the ADR + RZ group (*R* = 0.556). Importantly, a correlation was found between a lower freezing index in the FE task, suggesting better memory consolidation, with higher BDNF levels (*R* =  − 0.27 in ADR + RZ group and *R* =  − 0.71 in ADR + vehicle group; Suppl. Fig. S3). These data indicate that RZ augmented BDNF in the chemotherapy-exposed mice correlated with improved memory consolidation processes.

### Riluzole Reverses Chemotherapy-Induced Loss of Newly Born Neurons

**Fig. 4:**
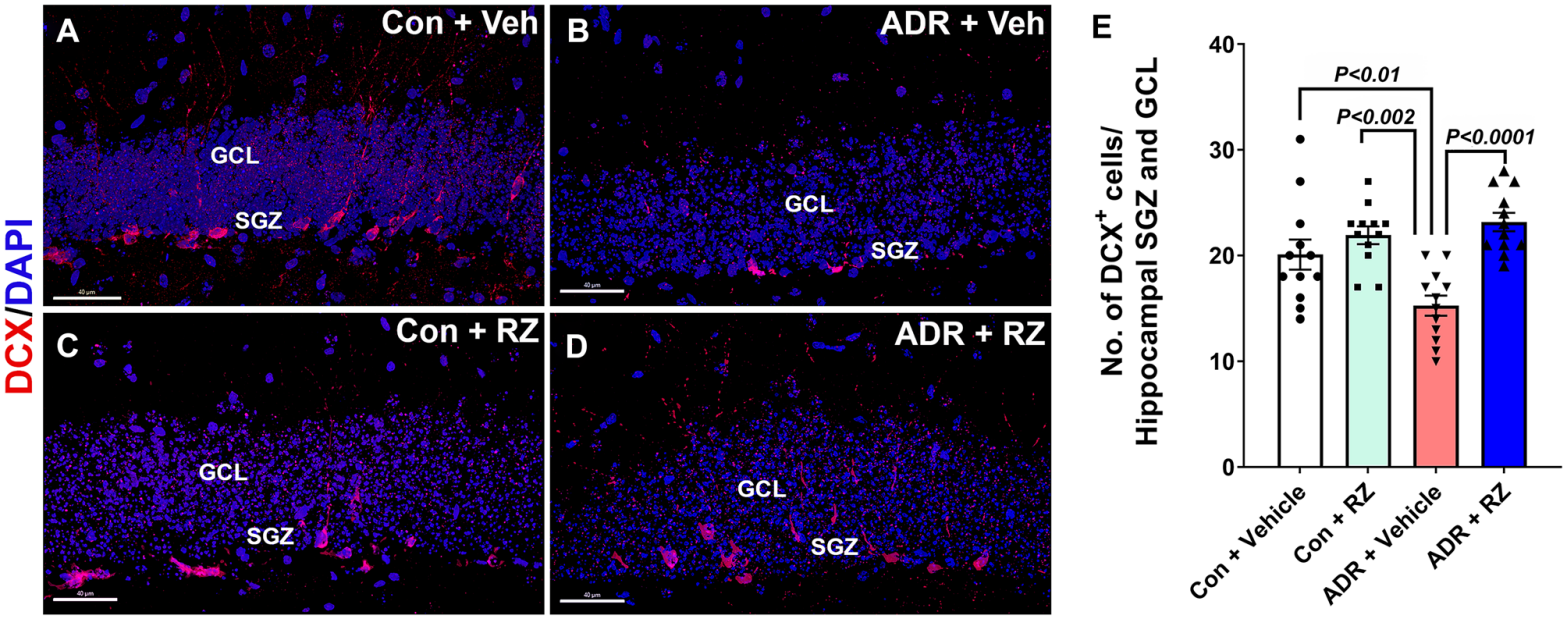
Riluzole treatment reverses chemotherapy-induced decline in newly born neurons. Adult female mice were treated with chronic ADR (2 mg/kg, i.p., once weekly for 4 weeks) and 24 h later received riluzole (RZ, 13 mg/kg) in drinking water for 6 to 7 weeks. **A-D. **Newly born, immature neurons (doublecortin, DCX) in the hippocampal sub-granular zone (SGZ) and granule cell layer (GCL) were assessed using immunofluorescence staining, confocal microscopy, and 3D algorithm-based unbiased spot analysis of DCX^+^ cells (red; DAPI, blue). **E. **ADR treatment significantly reduced the number of DCX^+^ neurons in the hippocampus compared with either control + vehicle or control + RZ groups. ADR-treated mice receiving RZ showed a significantly higher number of DCX^+^ cells compared with the ADR + vehicle group. Data are presented as mean ± SEM (*N* = 12 mice per group). *P* values were derived from a two-way ANOVA and Bonferroni’s multiple comparisons test. Scale bars, 40 μm, (**A**–**D**)

BDNF has been shown to play a critical role in maintaining neurogenesis and neuronal plasticity in the brain [38, 39]. In our past studies, we have shown that exposure to chronic chemotherapy adversely affects neurogenesis [29]. We assessed the impact of chronic ADR and RZ treatments on the expression of doublecortin (DCX), a surrogate marker of newly born, immature neurons in the hippocampal dentate gyrus. Following differentiation from neural stem cells, newly born neurons express DCX in the hippocampal granule cell layers (GCL) and sub-granular zone (SGZ) within 3 h and up to 12 to 14 days in vivo. We conducted immunofluorescence staining and quantification for DCX^+^ neurons in the hippocampal GCL and SGZ (Fig. 4A–D). The two-way ANOVA showed a significant RZ × ADR interaction (*F*_(1, 44)_ = 8.37, *P* = 0.006*)* and RZ effect (*F*_(1, 44)_ = 21.5, *P* < 0.0001) but no ADR effect (*F*_(1, 44)_ = 2.90, *P* = 0.10). The chronic ADR treatment (ADR + vehicle) led to a significant reduction in the number of DCX^+^ cells in hippocampal GCL and SGZ compared to either control + vehicle and control + RZ groups (Fig. 4E; *P* < 0.01 and *P* < 0.002, respectively). Treatment with RZ significantly increased the number of DCX^+^ cells in the ADR + RZ group compared with the ADR + vehicle group (*P* < 0.0001) indicating protective effects against chronic chemotherapy.

### Riluzole Reverses Chemotherapy-Induced Decline in Neurogenesis

**Fig. 5:**
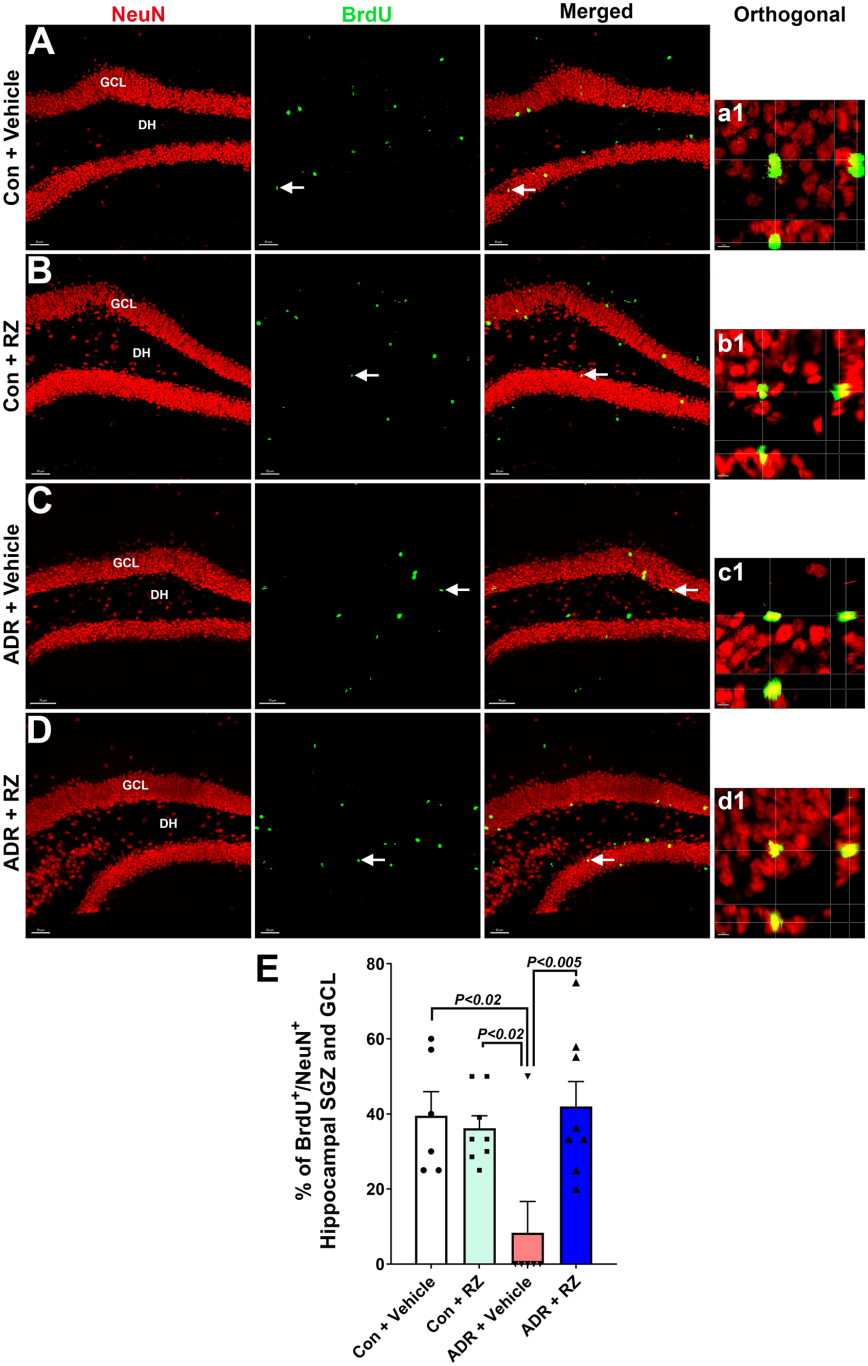
Riluzole treatment reverses chemotherapy-induced decline in neurogenesis. WT adult female mice treated with chronic ADR (2 mg/kg, i. p., once weekly for 4 weeks) received riluzole (RZ, 13 mg/kg) 24 h later in drinking water for 6 to 7 weeks. One week after the last ADR injection, mice were treated with BrdU, and hippocampal neurogenesis was quantified using BrdU-NeuN dual-immunofluorescence staining in the dentate gyrus 4 to 5 weeks after BrdU treatment. Compared to the control + vehicle (**A**, **a1**) and control + RZ (**B**, **b1**), ADR + vehicle mice (**C**, **c1**) showed a significant decline in neurogenesis, as indicated by the reduced numbers of the percentage of BrdU^+^ cells (green) differentiating into the mature neurons (red, NeuN; **C**, **c1**, **E**). RZ-treated mice retained significantly higher numbers of dual-fluorescent cells (BrdU^+^-NeuN^+^), similar to the control + vehicle group (**E**). Orthogonal z stacks (**a1**–**d1**) for the representative dual-labeled BrdU+ -NeuN+ cells (white arrows) are shown for each group (**A**–**D**). Data are presented as mean ± SEM (*N* = 6–8 mice per group). *P* values were derived from a two-way ANOVA and Bonferroni’s multiple comparisons test. GCL, granule cell layer; DH, dentate hilus. Scale bars, 50 μm, (**A**–**D**), and 5 μm, (**a1**–**d1**)

In the rodent hippocampus, after differentiation from neural stem cells, the maturation of newly born neurons takes approximately 26–27 days to express the mature neuronal marker NeuN. Next, we assessed the impact of RZ treatment and BDNF enhancement on the process of neurogenesis in vivo. One week after completion of chronic ADR treatment (Fig. 1A), female mice were treated with BrdU to label proliferating neural stem cells. The assessment of neurogenesis (BrdU^+^-NeuN^+^ dual-labeled cells) was conducted 7–8 weeks after BrdU injection (Fig. 5A–D, a1-d1). The two-way ANOVA analysis found a significant RZ × ADR interaction (*F*_(1, 24)_ = 8.78, *P* = 0.007*)* and RZ effect (*F*_(1, 24)_ = 5.89, *P* = 0.023*)* but no ADR effect (*F*_(1, 24)_ = 4.12.90, *P* = 0.06). ADR + vehicle groups showed a significantly reduced percentage of BrdU^+^-NeuN^+^ cells compared with control + vehicle and control + RZ groups (Fig. 5E, P < 0.02). Conversely, treatment with RZ (or augmentation of BDNF), significantly increased BrdU^+^-NeuN^+^ cells in the ADR + RZ group compared with the ADR + vehicle group (*P* < 0.005). This data corroborates our findings on regarding the numbers of immature neurons (Fig. 4, DCX) and asserts that RZ treatment is neuroprotective against chemotherapy-induced loss of neurogenesis.

### Riluzole Treatment Reduces Chemotherapy-Induced Neuroinflammation

**Fig. 6:**
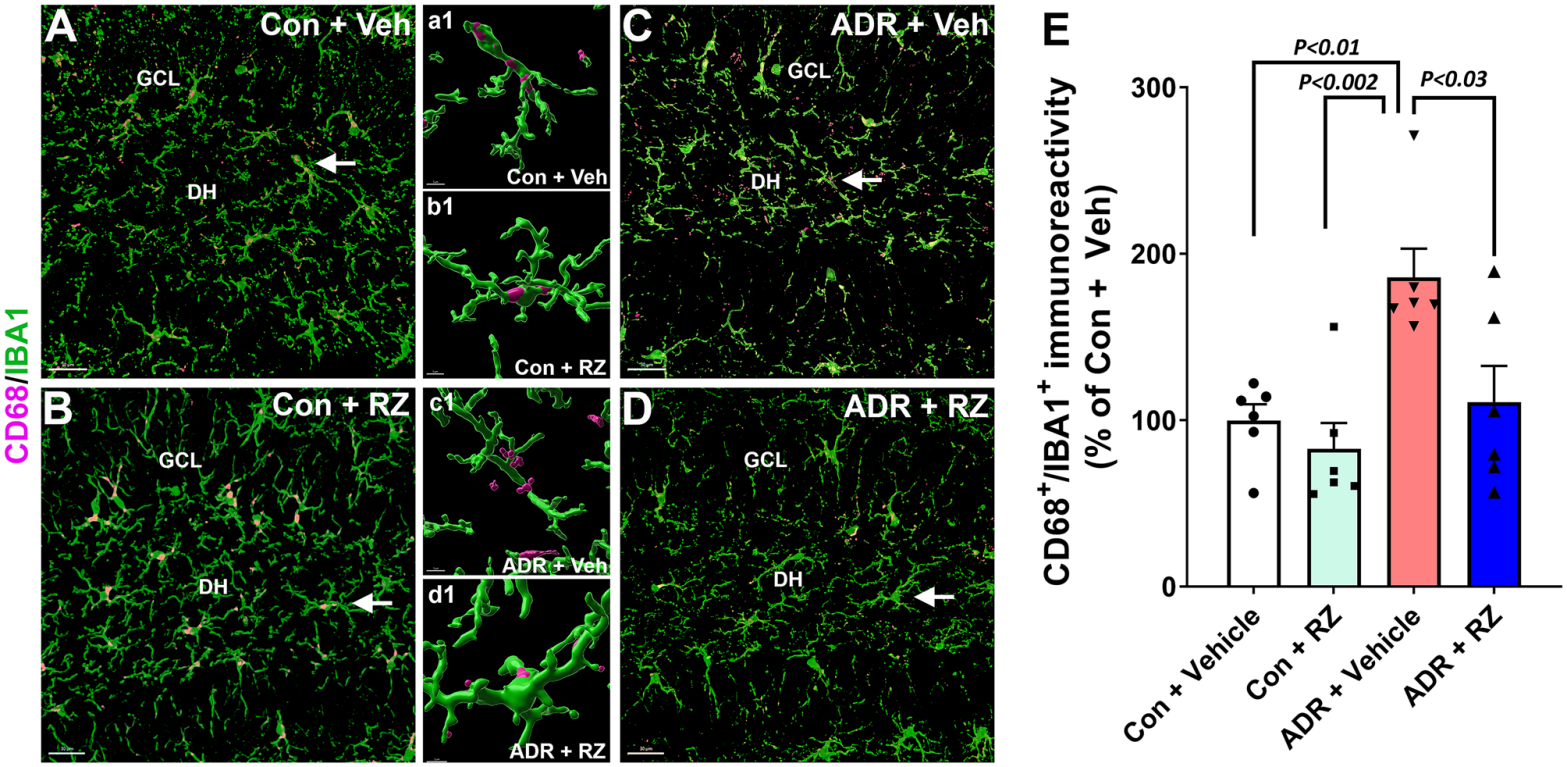
Riluzole treatment reduces chemotherapy-induced microglial activation. Adult female mice were treated with chronic ADR (2 mg/kg, i.p., once weekly for 4 weeks) and 24 h later received riluzole (RZ, 13 mg/kg) in drinking water for 6 to 7 weeks. Activated microglia in the hippocampal granule cell layer (GCL) and dentate hilus (DH) were assessed using dual immunofluorescence staining, laser scanning confocal microscopy, and 3D algorithm-based volumetric quantification for IBA1 (green) and CD68 (magenta) dual-labeling. **A**–**D** ADR treatment (ADR + vehicle group) significantly elevated the volume of activated microglia (CD68^+^-IBA1^+^) in the hippocampus compared with either control + vehicle or control + RZ groups. ADR-treated mice receiving RZ showed a significantly reduced volume of CD68^+^-IBA1^+^ co-labeling compared with the ADR + vehicle group. **a1**–**d1** Higher magnification surface reconstruction for the IBA1^+^ (green) positive microglia expressing CD68 puncta (magenta) is shown for the selected cells (white arrows, **A**–**D**) in each group. **E** Volumetric data for CD68^+^-IBA1^+^ co-expression is presented as mean ± SEM (*N* = 6 mice per group). *P* values were derived from a two-way ANOVA and Bonferroni’s multiple comparisons test. Scale bars, 30 μm, (**A**–**D**), and 5 μm, (**a1**–**d1**)

Our past studies have shown elevated neuroinflammation and microglial activation as one of the hallmarks in the rodent brains exposed to chronic chemotherapy [29, 37, 40]. To assess the effectiveness of RZ treatment on the status of microglial activation, dual immunofluorescence staining (Fig. 6A–D) and 3D algorithm-based volumetric analysis (Fig. 6E) were conducted on brain sections stained for a pan microglial marker and a lysosomal membrane protein marker representing microglial activation (IBA1 and CD68, respectively). We found elevated CD68 expression in the IBA1^+^ microglia (IBA1-CD68 co-labeling) in the brains of mice exposed to chronic ADR treatment (ADR + vehicle group). The two-way ANOVA revealed a significant RZ effect (*F*_(1, 20)_ = 7.64, *P* = 0.012) and ADR effect (*F*_(1, 20)_ = 11.68, *P* = 0.003) but no RZ × ADR interaction (*F*_(1, 20)_ = 2.99, *P* = 0.10). Chronic ADR treatment (ADR + vehicle) significantly increased CD68 immunoreactivity in the IBA1^+^ microglia compared to the control + vehicle and control + RZ groups (*P* = 0.01 and 0.002 respectively, Fig. 6E). Conversely, RZ treatment significantly reduced activated microglia in the ADR-treated brain compared to the ADR + vehicle group (*P* = 0.03). The quantification of IBA1 immunoreactivity alone did not reveal any significant differences between groups (Suppl. Fig. S4). These data provided a plausible link between the RZ treatment- and/or BDNF augmentation-mediated reduction in the microglial activation that reverses adverse effects of chemotherapy on the cognitive function.

## Discussion

This study provided pre-clinical evidence that RZ reverses chemotherapy-induced cognitive decline in a mouse model. Notably, in chemotherapy-exposed female mice, our data showed that treatment with RZ improves cognitive performance, along with other important observations signifying the reversal of biochemical and neurobiological underpinnings of chemotherapy-induced cognitive decline including elevated BDNF levels, neurogenesis, and neuroinflammation compared to vehicle-treated mice. Our results echoed the findings of other studies reported in the literature where treatment with RZ arrested cognitive decline in mouse models of AD, likely mediated through protection of hippocampal neurons [41, 42]. In cultured mouse astrocytes, RZ is shown to stimulate BDNF and glial cell line-derived neurotrophic factor (GDNF) synthesis [43].

Several human studies have reported a positive correlation between BDNF levels and improved cognitive function in cancer patients [44], supporting our hypothesis that augmenting BDNF has great potential to mitigate CRCI. Hence, the basis of this study stems from the breadth of our and others’ clinical data showing a positive correlation between the low BDNF levels with cognitive dysfunction in cancer patients receiving cytotoxic chemotherapy [3–7]. We postulated that the effects of chronic chemotherapy on BDNF expression could persist long after the completion of chemotherapy and in cancer survivors, resulting in the long-term CRCI [14]. In our past clinical studies [3, 12, 45], we consistently observed a statistically significant decrease in plasma BDNF levels at 6 and 12 weeks after breast cancer patients were treated with ADR and cyclophosphamide (ADR-CYP). This association between reduced BDNF and the higher risk of cognitive decline was still evident at 24 months post-treatment. These findings suggest that increasing or augmenting BDNF levels in vivo provides a therapeutic avenue to reverse short- and long-term CRCI.

It has been well established that along with other neurotrophins, BDNF plays a major role in cognitive performance, maintenance of neurogenesis, and neuronal plasticity in the brain [38, 39]. BDNF is highly expressed in the hippocampus, cortex, and basal forebrain and has an important role in regions that are vital to cognitive function, particularly hippocampal-dependent learning and memory [46]. BDNF plays a neuromodulatory role in the maintenance of LTP [10, 11, 39] that contribute in learning and memory consolidation [47]. Several studies have linked reductions in BDNF to the pathogenesis of cognitive disorders, such as AD, with low serum levels correlated with AD and mild cognitive impairment and high serum levels associated with better cognition in healthy older adults [8–11]. Our past pre-clinical studies have also shown a significant decline in newly born neurons (DCX^+^), hippocampal neurogenesis (BrdU-NeuN), and loss of neuronal structure and synaptic density that was accompanied by cognitive dysfunction following chronic ADR or CYP treatment [29, 37]. As chemotherapy diminishes dentate neurogenesis, it is plausible that it is partially linked to a decline in the hippocampal BDNF levels in the chemo-treated brains. In rat models of chemobrain, physical exercise prevented decline in newly born neurons, neurogenesis, apoptosis, and reduced cognitive impairments [48, 49]. Physical exercise or running has been shown to increase BDNF levels which is linked with improved synaptic plasticity, spinogenesis, dentate neurogenesis, and improved cognitive function in rodents [50]. Several human studies evaluated the role of non-pharmacological interventions such as cognitive training or integrative therapies to mitigate CRCI by increasing BDNF levels [44, 51–54]. Taken together, these pre-clinical and clinical studies supported our pharmacological approach to augment BDNF in vivo to protect from the loss of neurogenesis and thereby cognitive function following exposure to chemotherapy.

We have observed differences between the control + vehicle and control + RZ groups in Elevated Plus Maze (EPM). We have also observed significant differences between the control + vehicle and control + RZ groups in the extinction training days (days 2 and 3) of the FE task. After the completion of the extinction training phase (24 h after training day 3), the extinction test was administered (Fig. 2B), which serves as one of the benchmarks for the memory consolidation process, and we did not find any statistical significance (*P* = 0.95) between the control + vehicle and control + RZ groups. Importantly, for both the tasks, RZ treatment to the chemo-exposed mice was able to improve anxiety-like behavior (EPM) and memory consolidation deficits (FE). Furthermore, RZ treatment did not significantly increase the release of BDNF in healthy brains not exposed to chemotherapy. Albeit, EPM is one of the measures for anxiety-like behavior. We have also analyzed two other parameters to determine anxiety-like behavior, including open field activity (Suppl. Fig. S1) and total time exploring both novel and familiar locations in the NPR task (Suppl. Fig. S2). However, we did not find significant differences between the groups in total distance traveled (Fig. S1-A) and percentage time spent in the central zone (Fig. S1-B). Similarly, we did not find significant differences in total time spent exploring both objects in the NPR task, thereby excluding the possibility of neophobic behavior of mice in the open arena presented with objects. These observations indicate that control + RZ mice are not anxious per se, but it could be a task-specific behavior. The brain structural physiology and conductivities may differ between in mice exposed to chemotherapy and non-exposed to chemotherapy, which could also explain the differences observed. This is evident in other neurodegenerative conditions, such as Parkinson’s disease (PD), where BDNF expression is downregulated in the pars compacta of substantia niagra (SN) in the PD patients, which resulting in the deprivation of dopaminergic neurons from trophic support and the remaining dopaminergic neurons in SN produce progressively lower BDNF levels [55, 56]. In contrast, recurrent limbic seizures elevate BDNF which is linked with elevated neurogenesis and increased anterograde BDNF transport in the epileptic hippocampus [57]. The enhancement of BDNF by exogenous sources may be more obvious in the disease setting comparing to healthy settings. Although there was no statistically significant enhancement of BDNF levels after RZ administration in the control female mice (comparing control + vehicle vs. control + RZ), these results lack clinical relevance because it is unlikely to administer RZ to patients without exposure to chemotherapy or cancer. We have also observed the correlation of BDNF levels with freezing indices in the extinction test. Elevated freezing behavior was correlated with lower hippocampal BDNF levels in the ADR + vehicle group, and in contrast, lower freezing behavior (intact memory consolidation process) was correlated with higher BDNF in the hippocampus (Suppl. Fig. S3). Impaired fear extinction memory consolidation in the ADR + vehicle group indicates a disruption of neural processes involving multiple brain regions including hippocampus, mPFC, and amygdala [26, 58]. Such Pavlovian conditioning and dissociative learning involve long-term genetic, epigenetic, and synaptic remodeling [59, 60]. Importantly, the RZ treatment of the chemotherapy-exposed animals was able to restore memory consolidation indicating a long-term beneficial neuro-cognitive impact in the chemobrain.

Our previous pre-clinical studies [29, 37, 40] have also shown that chemotherapy can interfere with neurogenesis and elevate neuroinflammation that may culminate into cognitive dysfunction. Furthermore, clinical studies conducted by our group have observed a correlation between reduction of BDNF and increase in pro-inflammatory cytokines such as TNFα [45, 61]. Treatment with doxorubicin has also been shown to elevate plasma TNFα in rodents [62] that may disrupt the blood brain barrier integrity and aggravate neuroinflammation, including microglial activation. Increased levels of pro-inflammatory cytokines and neuroinflammation can adversely affect the downstream BDNF signaling [63]. In our study, we have observed that neural stem cells were protected from microglial activation and thus implying less reduction of neurogenesis in mice receiving RZ. Data from this study, along with our past pre-clinical and clinical studies, corroborate our current findings that in vivo enhancement of BDNF is one of the contributory neuroprotective factors in the mouse chemobrain model.

A number of mitigation strategies have been investigated to clinically manage CRCI, which include both non-pharmacological and pharmacological strategies [64, 65]. Unfortunately, the poor effectiveness for majority of the proposed intervention was likely due to the poor ability for these to target the underlying mechanisms associated with CRCI. Recent research has also evaluated whether novel modalities such as stem cells [66] or exosomes [67] are able to reverse CRCI. Hence, the utility of translationally feasible pharmacological strategy in the context of non-CNS cancer therapy has yet to be established and remains an area of limited research. Our strategy to augment BDNF in CRCI has also been trialed in a number of neurological complications such as AD [68], Parkinson’s disease [69], and depression [70]. In the AD, it has been shown that CREB-mediated transcription is down regulated by β-amyloid peptide in the amyloid plaques, which can contribute to a decrease of CREB-regulated BDNF levels. Non-pharmacological strategies such as exercise can improve learning and memory impairment by upregulating the BDNF pathway in animal models of AD [68] and in Parkinson’s disease [69]. Investigators have also evaluated the efficacy of utilizing Mediterranean diet to improvement plasma BDNF levels in patients diagnosed with depression [70]. It is important to note that current interventions are mostly focused on non-pharmacological strategies, with a lack of pharmacological agents demonstrating the effectiveness to improve disease as well as associated BDNF levels.

We acknowledge that in addition to protection of neurogenesis, RZ-mediated beneficial neuroprotective effects in the chemobrain model are likely mediated through other neuronal mechanisms including maintenance of neuronal plasticity or LTP, protection of dendritic structure, spine density, and synapses. For example, AD mice treated with RZ had long-term memory comparable to the age-matched controls that was hypothesized to be mediated through reducing glutamate release during early AD as glutaminergic hyperactivity is known to cause a neurotoxic effect on hippocampal neurons [41, 42]. The eventual goal of our approach is to test this pharmacologic strategy in a clinically relevant, cancer-bearing model receiving combination chemo- or radiation-therapy to broaden its prospects to ameliorate CRCI. Based on the results of our study, we posit that the use of RZ is likely most beneficial in patients who are receiving chemotherapy (such as ADR) who are at high risk for CRCI. However, before RZ can be utilized in the clinical setting, it is vital to ensure that RZ does not interfere with chemotherapy, and the safety/efficacy of RZ needs to be tested in the pre-clinical mouse models of cancer receiving chemotherapy to recapitulate clinical scenario.

## Conclusion

In summary, our data provides pre-clinical evidence for a translationally feasible pharmacological approach, oral delivery of riluzole, to augment BDNF in vivo to mitigate cancer chemotherapy-induced adverse impact on neurogenesis, microglial activation, and cognitive function.

## Supplementary Information

Below is the link to the electronic supplementary material.

Supplementary file1 (PDF 559 kb)

Supplementary file2 (PDF 556 kb)

Supplementary file3 (PDF 568 kb)

Supplementary file4 (PDF 516 kb)

Supplementary file5 (PDF 533 kb)

Supplementary file6 (PDF 515 kb)

Supplementary file7 (PDF 551 kb)

Supplementary file8 (PDF 525 kb)

Supplementary file9 (PDF 542 kb)

Supplementary file10 (PDF 306 kb)

## Data Availability

All data and material are available per request.

## References

[1] Cheung YT, Shwe M, Tan YP, Fan G, Ng R, Chan A (2012). Cognitive changes in multiethnic Asian breast cancer patients: a focus group study. Ann Oncol.

[2] Ng T, Dorajoo SR, Cheung YT, Lam YC, Yeo HL, Shwe M (2018). Distinct and heterogeneous trajectories of self-perceived cognitive impairment among Asian breast cancer survivors. Psychooncology.

[3] Yap NY, Tan NYT, Tan CJ, Loh KW, Ng RCH, Ho HK (2020). Associations of plasma brain-derived neurotrophic factor (BDNF) and Val66Met polymorphism (rs6265) with long-term cancer-related cognitive impairment in survivors of breast cancer. Breast Cancer Res Treat.

[4] Bury-Kaminska M, Szudy-Szczyrek A, Nowaczynska A, Jankowska-Lecka O, Hus M, Kot K. Chemotherapy-related differences in cognitive functioning and their biological predictors in patients with multiple myeloma. Brain Sci. 2021;11(9). 10.3390/brainsci11091166 PMC8466339 34573187

[5] Jehn CF, Becker B, Flath B, Nogai H, Vuong L, Schmid P (2015). Neurocognitive function, brain-derived neurotrophic factor (BDNF) and IL-6 levels in cancer patients with depression. J Neuroimmunol.

[6] Zimmer P, Mierau A, Bloch W, Struder HK, Hulsdunker T, Schenk A (2015). Post-chemotherapy cognitive impairment in patients with B-cell non-Hodgkin lymphoma: a first comprehensive approach to determine cognitive impairments after treatment with rituximab, cyclophosphamide, doxorubicin, vincristine and prednisone or rituximab and bendamustine. Leuk Lymphoma.

[7] Guo JC, Yang YJ, Zheng JF, Guo M, Wang XD, Gao YS (2019). Functional rs6265 polymorphism in the brain-derived neurotrophic factor gene confers protection against neurocognitive dysfunction in posttraumatic stress disorder among Chinese patients with hepatocellular carcinoma. J Cell Biochem.

[8] Gunstad J, Benitez A, Smith J, Glickman E, Spitznagel MB, Alexander T (2008). Serum brain-derived neurotrophic factor is associated with cognitive function in healthy older adults. J Geriatr Psychiatry Neurol.

[9] Shimada H, Makizako H, Doi T, Yoshida D, Tsutsumimoto K, Anan Y (2014). A large, cross-sectional observational study of serum BDNF, cognitive function, and mild cognitive impairment in the elderly. Front Aging Neurosci.

[10] Teixeira AL, Barbosa IG, Diniz BS, Kummer A (2010). Circulating levels of brain-derived neurotrophic factor: correlation with mood, cognition and motor function. Biomark Med.

[11] Zhang XY, Chen DC, Xiu MH, Haile CN, Luo X, Xu K (2012). Cognitive and serum BDNF correlates of BDNF Val66Met gene polymorphism in patients with schizophrenia and normal controls. Hum Genet.

[12] Ng T, Lee YY, Chae JW, Yeo AHL, Shwe M, Gan YX (2017). Evaluation of plasma brain-derived neurotrophic factor levels and self-perceived cognitive impairment post-chemotherapy: a longitudinal study. BMC Cancer.

[13] Chan A, Cheng I, Wang C, Tan CJ, Toh YL, Ng DQ, et al. Cognitive impairment in adolescent and young adult cancer patients: Pre-treatment findings of a longitudinal study. Cancer Med. 2022. 10.1002/cam4.5295 PMC9972136 36221816

[14] Alhowail AH, Bloemer J, Majrashi M, Pinky PD, Bhattacharya S, Yongli Z (2019). Doxorubicin-induced neurotoxicity is associated with acute alterations in synaptic plasticity, apoptosis, and lipid peroxidation. Toxicol Mech Methods.

[15] Molinari C, Morsanuto V, Ruga S, Notte F, Farghali M, Galla R, et al. The Role of BDNF on Aging-Modulation Markers. Brain Sci. 2020;10(5). 10.3390/brainsci10050285 PMC7287884 32397504

[16] Nagahara AH, Tuszynski MH (2011). Potential therapeutic uses of BDNF in neurological and psychiatric disorders. Nat Rev Drug Discov.

[17] Katoh-Semba R, Asano T, Ueda H, Morishita R, Takeuchi IK, Inaguma Y (2002). Riluzole enhances expression of brain-derived neurotrophic factor with consequent proliferation of granule precursor cells in the rat hippocampus. FASEB J.

[18] Hunsberger HC, Weitzner DS, Rudy CC, Hickman JE, Libell EM, Speer RR (2015). Riluzole rescues glutamate alterations, cognitive deficits, and tau pathology associated with P301L tau expression. J Neurochem.

[19] Gourley SL, Espitia JW, Sanacora G, Taylor JR (2012). Antidepressant-like properties of oral riluzole and utility of incentive disengagement models of depression in mice. Psychopharmacology.

[20] Okamoto M, Gray JD, Larson CS, Kazim SF, Soya H, McEwen BS (2018). Riluzole reduces amyloid beta pathology, improves memory, and restores gene expression changes in a transgenic mouse model of early-onset Alzheimer's disease. Transl Psychiatry.

[21] Chao OY, de Souza Silva MA, Yang YM, Huston JP (2020). The medial prefrontal cortex - hippocampus circuit that integrates information of object, place and time to construct episodic memory in rodents: Behavioral, anatomical and neurochemical properties. Neurosci Biobehav Rev.

[22] Barker GR, Bird F, Alexander V, Warburton EC (2007). Recognition memory for objects, place, and temporal order: a disconnection analysis of the role of the medial prefrontal cortex and perirhinal cortex. J Neurosci.

[23] Barker GR, Warburton EC (2011). When is the hippocampus involved in recognition memory?. J Neurosci.

[24] Walf AA, Frye CA (2007). The use of the elevated plus maze as an assay of anxiety-related behavior in rodents. Nat Protoc.

[25] Cain CK, Blouin AM, Barad M (2003). Temporally massed CS presentations generate more fear extinction than spaced presentations. J Exp Psychol Anim Behav Process.

[26] Chang CH, Knapska E, Orsini CA, Rabinak CA, Zimmerman JM, Maren S. Fear extinction in rodents. Curr Protoc Neurosci. 2009;Chapter 8:Unit8.23. 10.1002/0471142301.ns0823s47 PMC2756523 19340814

[27] Johansen JP, Cain CK, Ostroff LE, LeDoux JE (2011). Molecular mechanisms of fear learning and memory. Cell.

[28] Baulch JE, Acharya MM, Allen BD, Ru N, Chmielewski NN, Martirosian V (2016). Cranial grafting of stem cell-derived microvesicles improves cognition and reduces neuropathology in the irradiated brain. Proc Natl Acad Sci U S A.

[29] Christie LA, Acharya MM, Parihar VK, Nguyen A, Martirosian V, Limoli CL (2012). Impaired cognitive function and hippocampal neurogenesis following cancer chemotherapy. Clin Cancer Res.

[30] Acharya MM, Baddour AA, Kawashita T, Allen BD, Syage AR, Nguyen TH (2017). Epigenetic determinants of space radiation-induced cognitive dysfunction. Sci Rep.

[31] Acharya MM, Baulch JE, Lusardi TA, Allen BD, Chmielewski NN, Baddour AA (2016). Adenosine kinase inhibition protects against cranial radiation-induced cognitive dysfunction. Front Mol Neurosci.

[32] Acharya MM, Green KN, Allen BD, Najafi AR, Syage A, Minasyan H (2016). Elimination of microglia improves cognitive function following cranial irradiation. Sci Rep.

[33] Markarian M, Krattli RP, Baddour JD, Alikhani L, Giedzinski E, Usmani MT (2021). Glia-selective deletion of complement C1q prevents radiation-induced cognitive deficits and neuroinflammation. Cancer Res.

[34] Smith SM, Giedzinski E, Angulo MC, Lui T, Lu C, Park AL (2020). Functional equivalence of stem cell and stem cell-derived extracellular vesicle transplantation to repair the irradiated brain. Stem Cells Transl Med.

[35] Lueptow LM. Novel Object Recognition Test for the Investigation of Learning and Memory in Mice. J Vis Exp. 2017;(126). 10.3791/55718 PMC5614391 28892027

[36] Curzon P, Rustay NR, Browman KE. Cued and contextual fear conditioning for rodents. In: JJ B, editor. Methods of behavior analysis in neuroscience. Boca Raton (FL): CRC Press/Taylor & Francis; 2009. 21204331

[37] Acharya MM, Martirosian V, Chmielewski NN, Hanna N, Tran KK, Liao AC (2015). Stem cell transplantation reverses chemotherapy-induced cognitive dysfunction. Cancer Res.

[38] Huang EJ, Reichardt LF (2001). Neurotrophins: roles in neuronal development and function. Annu Rev Neurosci.

[39] Leal G, Bramham CR, Duarte CB (2017). BDNF and hippocampal synaptic plasticity. Vitam Horm.

[40] Allen BD, Apodaca LA, Syage AR, Markarian M, Baddour AAD, Minasyan H (2019). Attenuation of neuroinflammation reverses Adriamycin-induced cognitive impairments. Acta Neuropathol Commun.

[41] Hascup KN, Findley CA, Britz J, Esperant-Hilaire N, Broderick SO, Delfino K, et al. Riluzole attenuates glutamatergic tone and cognitive decline in AbetaPP/PS1 mice. J Neurochem. 2020. 10.1111/jnc.15224 PMC7902360 33107040

[42] Lesuis SL, Kaplick PM, Lucassen PJ, Krugers HJ (2019). Treatment with the glutamate modulator riluzole prevents early life stress-induced cognitive deficits and impairments in synaptic plasticity in APPswe/PS1dE9 mice. Neuropharmacology.

[43] Mizuta I, Ohta M, Ohta K, Nishimura M, Mizuta E, Kuno S (2001). Riluzole stimulates nerve growth factor, brain-derived neurotrophic factor and glial cell line-derived neurotrophic factor synthesis in cultured mouse astrocytes. Neurosci Lett.

[44] Ng DQ, Chan D, Agrawal P, Zhao W, Xu X, Acharya M (2022). Evidence of brain-derived neurotrophic factor in ameliorating cancer-related cognitive impairment: A systematic review of human studies. Crit Rev Oncol Hematol.

[45] Yap NY, Toh YL, Tan CJ, Acharya MM, Chan A (2021). Relationship between cytokines and brain-derived neurotrophic factor (BDNF) in trajectories of cancer-related cognitive impairment. Cytokine.

[46] Lu B, Nagappan G, Lu Y (2014). BDNF and synaptic plasticity, cognitive function, and dysfunction. Handb Exp Pharmacol.

[47] Morris RG, Anderson E, Lynch GS, Baudry M (1986). Selective impairment of learning and blockade of long-term potentiation by an N-methyl-D-aspartate receptor antagonist, AP5. Nature.

[48] Park HS, Kim CJ, Kwak HB, No MH, Heo JW, Kim TW (2018). Physical exercise prevents cognitive impairment by enhancing hippocampal neuroplasticity and mitochondrial function in doxorubicin-induced chemobrain. Neuropharmacology.

[49] Winocur G, Wojtowicz JM, Huang J, Tannock IF (2014). Physical exercise prevents suppression of hippocampal neurogenesis and reduces cognitive impairment in chemotherapy-treated rats. Psychopharmacology.

[50] Vivar C, Potter MC, van Praag H (2013). All about running: synaptic plasticity, growth factors and adult hippocampal neurogenesis. Curr Top Behav Neurosci.

[51] Gooch M, Mehta A, John T, Lomeli N, Naeem E, Mucci G, et al. Feasibility of cognitive training to promote recovery in cancer-related cognitive impairment in adolescent and young adult patients. J Adolesc Young Adult Oncol. 2021. 10.1089/jayao.2021.0055 PMC9464087 34672806

[52] Palmer ACS, Zortea M, Souza A, Santos V, Biazus JV, Torres ILS (2020). Clinical impact of melatonin on breast cancer patients undergoing chemotherapy; effects on cognition, sleep and depressive symptoms: A randomized, double-blind, placebo-controlled trial. PLoS ONE.

[53] Tong T, Pei C, Chen J, Lv Q, Zhang F, Cheng Z (2018). Efficacy of acupuncture therapy for chemotherapy-related cognitive impairment in breast cancer patients. Med Sci Monit.

[54] Hartman SJ, Weiner LS, Nelson SH, Natarajan L, Patterson RE, Palmer BW (2019). Mediators of a physical activity intervention on cognition in breast cancer survivors: evidence from a randomized controlled trial. JMIR Cancer.

[55] Baquet ZC, Bickford PC, Jones KR (2005). Brain-derived neurotrophic factor is required for the establishment of the proper number of dopaminergic neurons in the substantia nigra pars compacta. J Neurosci.

[56] Rahmani F, Saghazadeh A, Rahmani M, Teixeira AL, Rezaei N, Aghamollaii V (2019). Plasma levels of brain-derived neurotrophic factor in patients with Parkinson disease: a systematic review and meta-analysis. Brain Res.

[57] Binder DK, Croll SD, Gall CM, Scharfman HE (2001). BDNF and epilepsy: too much of a good thing?. Trends Neurosci.

[58] Quirk GJ, Mueller D (2008). Neural mechanisms of extinction learning and retrieval. Neuropsychopharmacology.

[59] Kwapis JL, Alaghband Y, Lopez AJ, White AO, Campbell RR, Dang RT (2017). Context and auditory fear are differentially regulated by HDAC3 activity in the lateral and basal subnuclei of the amygdala. Neuropsychopharmacology.

[60] Marshall PR, Bredy TW (2019). Neuroepigenetic mechanisms underlying fear extinction: emerging concepts. Psychopharmacology.

[61] Oppegaard K, Harris CS, Shin J, Paul SM, Cooper BA, Chan A (2021). Cancer-related cognitive impairment is associated with perturbations in inflammatory pathways. Cytokine.

[62] Tangpong J, Cole MP, Sultana R, Joshi G, Estus S, Vore M (2006). Adriamycin-induced, TNF-alpha-mediated central nervous system toxicity. Neurobiol Dis.

[63] Lima Giacobbo B, Doorduin J, Klein HC, Dierckx R, Bromberg E, de Vries EFJ (2019). Brain-derived neurotrophic factor in brain disorders: focus on neuroinflammation. Mol Neurobiol.

[64] Karschnia P, Parsons MW, Dietrich J (2019). Pharmacologic management of cognitive impairment induced by cancer therapy. Lancet Oncol.

[65] Mayo SJ, Lustberg M, Dhillon HM, Nakamura ZM, Allen DH, Von Ah D, et al. Cancer-related cognitive impairment in patients with non-central nervous system malignancies: an overview for oncology providers from the MASCC Neurological Complications Study Group. Support Care Cancer. 2020. 10.1007/s00520-020-05860-9 33231809

[66] Boukelmoune N, Laumet G, Tang Y, Ma J, Mahant I, Singh SK (2021). Nasal administration of mesenchymal stem cells reverses chemotherapy-induced peripheral neuropathy in mice. Brain Behav Immun.

[67] Koh YQ, Tan CJ, Toh YL, Sze SK, Ho HK, Limoli CL, et al. Role of exosomes in cancer-related cognitive impairment. Int J Mol Sci. 2020;21(8). 10.3390/ijms21082755 PMC7215650 32326653

[68] Azimi M, Gharakhanlou R, Naghdi N, Khodadadi D, Heysieattalab S (2018). Moderate treadmill exercise ameliorates amyloid-beta-induced learning and memory impairment, possibly via increasing AMPK activity and up-regulation of the PGC-1alpha/FNDC5/BDNF pathway. Peptides.

[69] Palasz E, Wysocka A, Gasiorowska A, Chalimoniuk M, Niewiadomski W, Niewiadomska G. BDNF as a promising therapeutic agent in Parkinson's disease. Int J Mol Sci. 2020;21(3). 10.3390/ijms21031170 PMC7037114 32050617

[70] Sanchez-Villegas A, Galbete C, Martinez-Gonzalez MA, Martinez JA, Razquin C, Salas-Salvado J (2011). The effect of the Mediterranean diet on plasma brain-derived neurotrophic factor (BDNF) levels: the PREDIMED-NAVARRA randomized trial. Nutr Neurosci.

