# Correction to: BDNF Augmentation Using Riluzole Reverses Doxorubicin-Induced Decline in Cognitive Function and Neurogenesis

**DOI:** 10.1007/s13311-023-01381-5

**Published:** 2023-05-03

**Authors:** Manal T. Usmani, Robert P. Krattli, Sanad M. El-Khatib, Anh C. D. Le, Sarah M. Smith, Janet E. Baulch, Ding Quan Ng, Munjal M. Acharya, Alexandre Chan

**Affiliations:** 1 Department of Anatomy and Neurobiology, School of Medicine, University of California, Irvine, CA USA; 2 Department of Radiation Oncology, School of Medicine, University of California, Irvine, CA USA; 3 Department of Clinical Pharmacy Practice, School of Pharmacy & Pharmaceutical Sciences, University of California, Irvine, CA USA; 4 Department of Pharmaceutical Sciences, School of Pharmacy and Pharmaceutical Sciences, University of California, Irvine, CA USA

**Correction to: Neurotherapeutics** **https://doi.org/10.1007/s13311-022-01339-z**

The following correction should be noted to the caption for Fig. 5 in the article as original published:

"...the percentage of BrdU+ cells (red) differentiating into the mature neurons (green, NeuN; **C, c1, E**)."

should have read

"...the percentage of BrdU+ cells (green) differentiating into the mature neurons (red, NeuN; **C, c1, E**)."

The original article has been corrected.

