# Supplementary material for: BDNF Augmentation Using Riluzole Reverses Doxorubicin-Induced Decline in Cognitive Function and Neurogenesis
Source: Neurotherapeutics. 2023 Jan 31;20(3):838–52. doi: 10.1007/s13311-022-01339-z (PMC10275819; doi:10.1007/s13311-022-01339-z)
Supplement: Supplementary file 10 — Supplementary file10 (PDF 306 kb) [file 13311_2022_1339_MOESM10_ESM.pdf]

SUPPLEMENTAL INFORMATION

BDNF augmentation using riluzole reverses doxorubicin-induced decline in cognitive function and neurogenesis. Usmani *et al.*, 2023. *Neurotherapeutics*, https://doi.org/10.1007/s13311-022-01339-z.

Supplemental Figure S1:

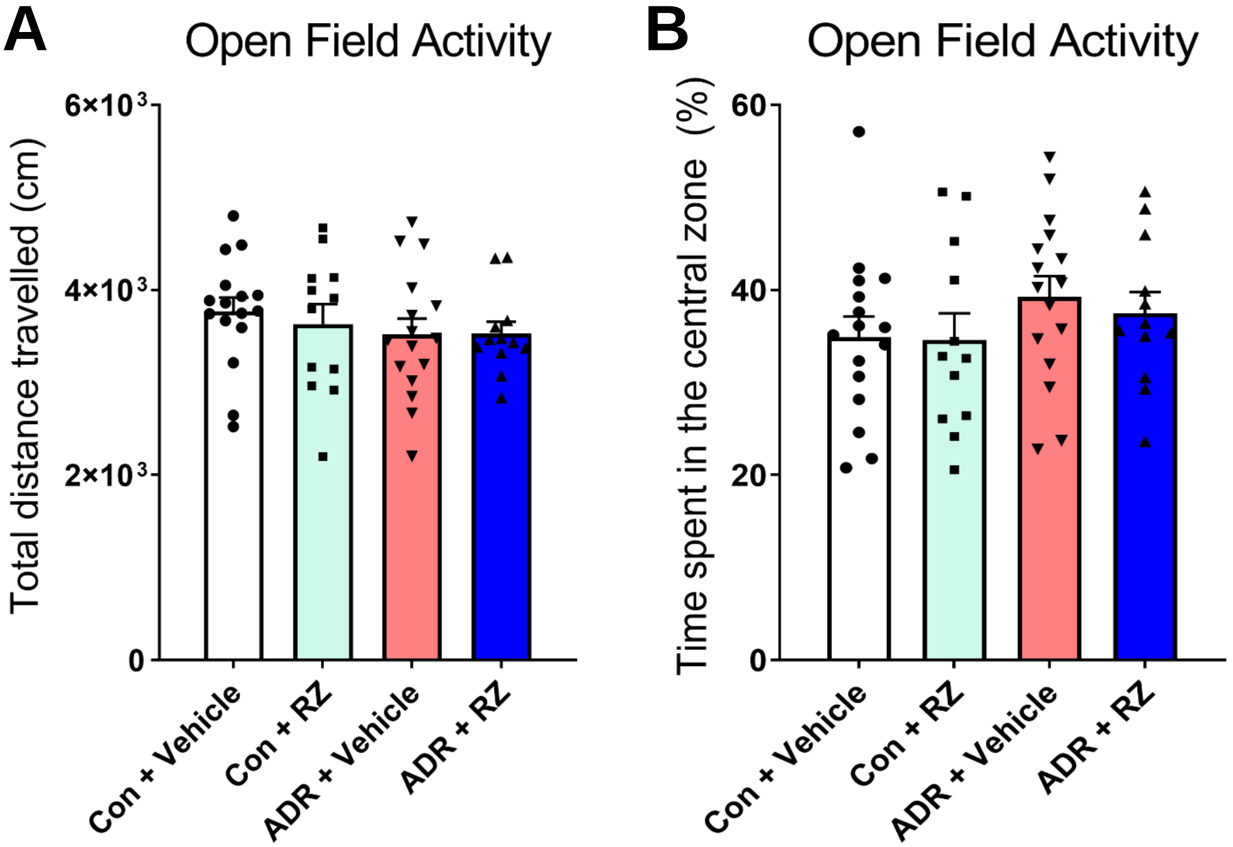

Supplemental Figure S1: Treatment with chronic ADR or riluzole did not affect the open field activity of animals in open arenas. All groups of mice were handled by the experimenter for one week. On day 1 of the habituation phase for the novel place recognition task (no objects), the total distance traveled (**A**) and percentage time spent in the central zone (**B**, 60% area) did not differ between the experimental group indicating the absence of the neophobic behavior. Data are presented as mean ± SEM (*N* =12-16 mice per group).

Supplemental Figure S2:

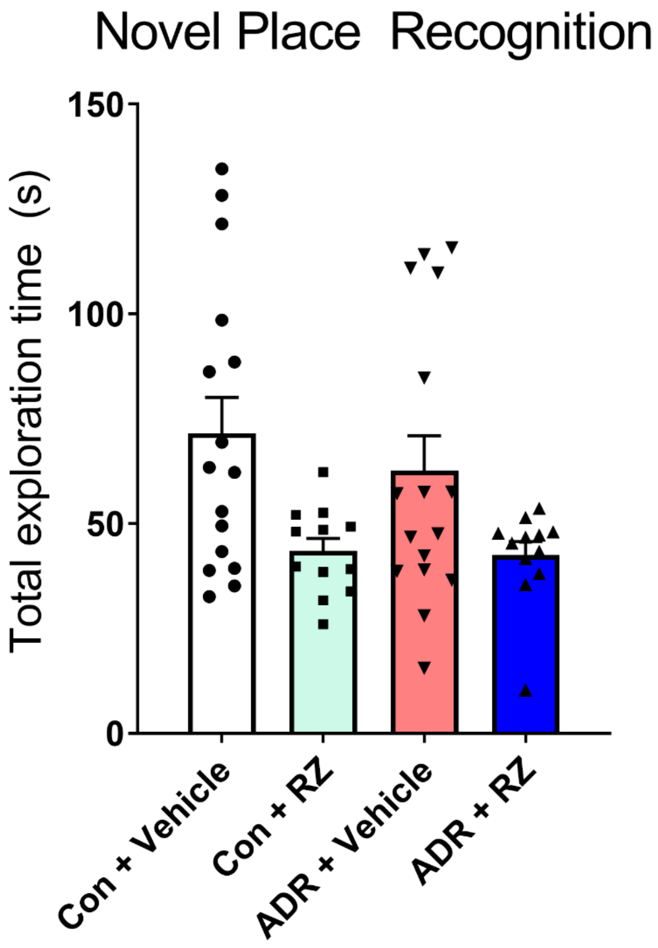

Supplemental Figure S2. Treatment with chronic ADR or riluzole did not affect total exploration time in the novel place recognition (NPR) task. All groups of mice were handled by the experimenter for one week and habituated in open arenas for three days prior to the NPR test. The total time exploring both familiar and novel placements of object did not differ between the experimental groups in the NPR test. Data are presented as mean ± SEM (*N* =12-16 mice per group).

Supplemental Figure S3:

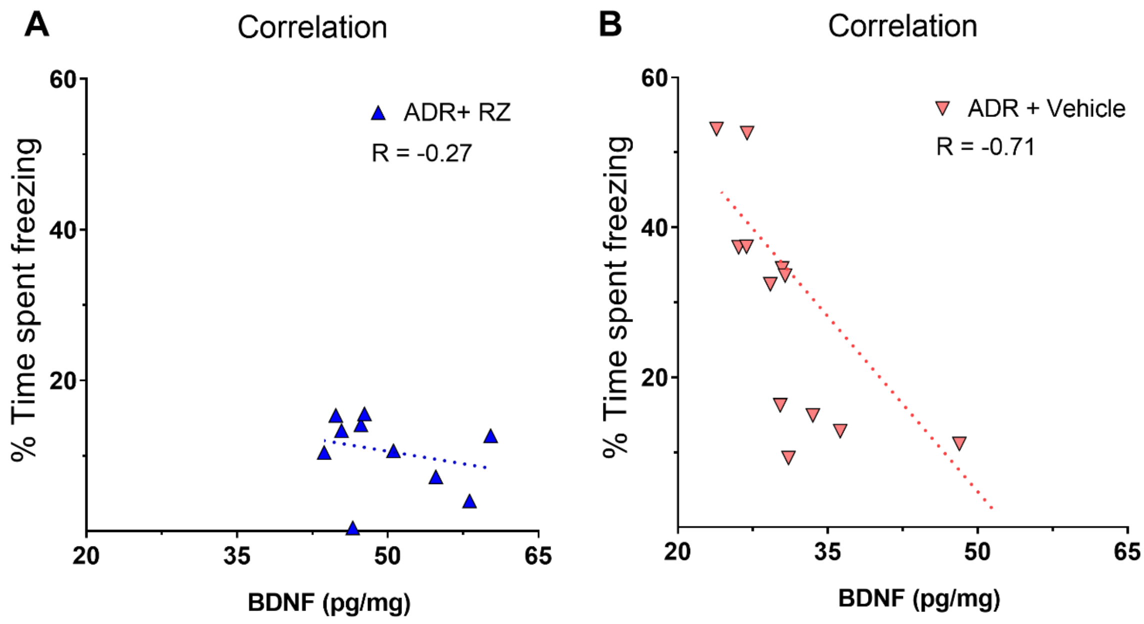

Supplemental Figure S3. Lower freezing indices in the extinction test (intact memory consolidation) in the (**A**) ADR + RZ group were correlated with higher BDNF levels and vice versa for the (**B**) ADR + Vehicle group. Correlation between BDNF levels with discrimination index (DI, NPR task) was inconclusive.

Supplemental Figure S4.

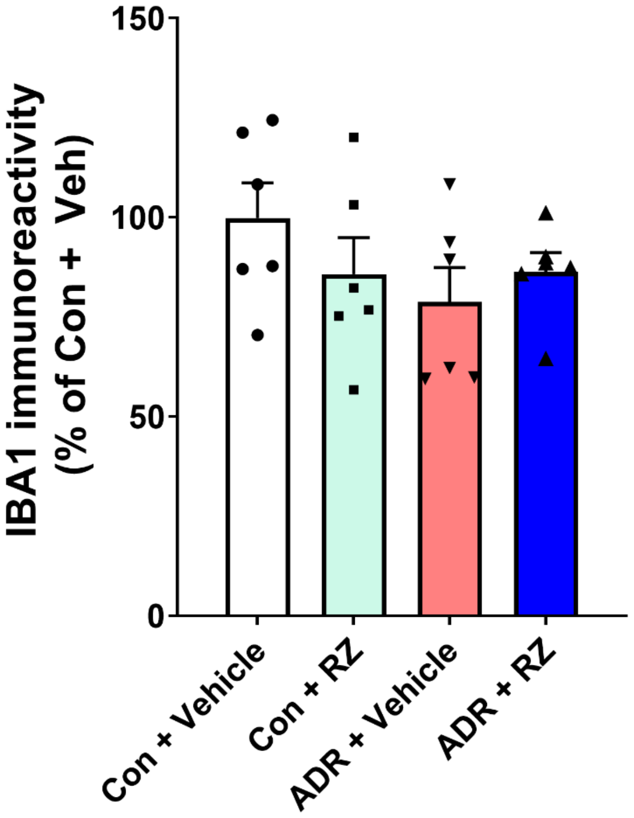

Supplemental Figure S4. Treatment with chronic ADR or riluzole did not affect IBA1$^+$ microglial immunoreactivity. IBA1 immunostaining, laser scanning confocal microscopy and 3D algorithm-based volumetric quantification of IBA1$^+$ surfaces (as in Fig. 6) were conducted as described in the Methods. Volumetric reconstruction of the IBA1$^+$ surfaces did not differ between the experimental group. Data are presented as mean ± SEM (*N* =6 mice per group).

MATERIALS AND METHODS

Cognitive testing

Novel place recognition task: One month after the initiation of RZ treatment, all the groups of mice were administered the NPR task. The performance on the NPR task depends on animals' inherent ability to explore novel object locations and rely on the intact hippocampal function. Briefly, mice were habituated for 3 days (15 min per day) to a dimly lit (48 lux) custom-made empty arena (30 × 30 × 30 cm) with a layer of fresh corncob bedding and recorded using a ceiling-mounted digital USB COMS camera (Noldus Information Technology). The open field activity was recorded for day 1 of habituation without any objects present (as shown in **Suppl. Fig. 1**). In between familiarization and test phases, all bedding was replaced and the arena was thoroughly cleaned with 10% ethanol. On the testing day, two plastic objects (Duplo blocks, similar size, and shape) were magnetically affixed 16 cm apart in the arena and the mouse was allowed 5 min to explore the objects. The mouse was then returned to the home cage for 5 min while the location of one object was moved to a novel spatial location after cleaning the object using 10% ethanol. The mouse was then returned to the arena for 5 min of further exploration. The total exploration time for animals exploring both the objects was recorded during the test phase (as shown in **Suppl. Fig. 2**). All trials were scored by the Noldus EthoVision XT (v16.0) automated multipoint animal tracking module. Time spent interacting (nose within 2 cm radius) with familiar versus novel object location was recorded and the data was calculated as the discrimination index (DI): ([Novel location exploration time/Total exploration time] – [Familiar location exploration time/Total exploration time]) × 100.

Elevated plus maze: The impact of chemotherapy and/or RZ treatment on anxiety-like behavior was evaluated using the elevated plus maze (EPM) task. This task provides a measure of exploratory behavior and anxiety by assessing the amount of time individual animals spend in two brightly-lit, open arms (~1300 lux) versus two closed, dark arms (~500 lux) on a maze elevated from the floor. The EPM was raised about 45 inches from the floor and consists of 45 x 45 inches of arms (Med Associates). Animals were placed in the central zone of the maze and allowed to explore freely for 5 mins. In between the individual animals, the maze was cleaned using non-alcoholic, detergent spray and dried. The exploration was monitored, tracked, and evaluated using the ceiling-mounted camera connected to the Noldus Ethovision XT module (v16.0).

Fear extinction memory consolidation task: To determine if chronic chemotherapy or RZ treatment affects amygdala-hippocampal circuit-dependent fear conditioning learning and fear memory consolidation, we performed fear extinction behavior reliant on hippocampal function. Testing occurred in a behavioral conditioning chamber (17.5 × 17.5 × 18 cm, Coulbourn Instruments) with steel shock floors (3.2 mm diameter slats, 8 mm spacing). Throughout the conditioning, memory consolidation (extinction training) and extinction testing phases, the bottom acrylic collection plate was scented with a spray of 10% acetic acid in water. For the initial fear conditioning phase, mice were allowed to habituate to the chamber for two minutes. Three pairings of an auditory conditioned stimulus (16 kHz tone, 80 dB, lasting 120 sec; CS) co-terminating with a foot shock unconditioned stimulus (0.6 mA, 1 sec; US) were presented at two-minute intervals. On the following three days of extinction training phase (Days 1-3), mice were initially habituated to the same context for two minutes before being presented with 20 non-US reinforced CS tones (16 kHz, 80 dB, lasting 120 sec, at 5-sec intervals). On a final day of fear testing mice were presented with only three non-US reinforced CS tones (16 kHz, 80 dB, lasting 120 sec) at two-minute intervals in the same context. Freezing behavior was recorded with a camera mounted above the chamber and scored by an automated measurement program (FreezeFrame, Coulbourn Instruments). FreezeFrame algorithms calculate a motion index for each frame of the video, with higher values representing greater motion. An investigator blinded to the experimental groups set the motion index threshold representing immobility for each animal individually, based on identifying a trough separating low values during immobility and higher values associated with motion. Freezing behavior was defined as continuous bouts of one second or more of immobility. The percentage of time each mouse spent freezing during the tone was then calculated for the conditioning, extinction training (average of five tones, four data points per day), and testing phases.

Immunohistochemistry, confocal microscopy, and *in silico* volumetric quantification

After completion of cognitive function tests, mice were euthanized (intra-cardiac perfusion) using saline with heparin (10U/ml, Sigma) and 4% PFA made in 100 mM PBS, pH 7.4 (paraformaldehyde, phosphate-buffered saline, Sigma). Brains were fixed overnight at 4 ºC in 4% PFA. Tissues were then cryo-protected (10 to 30% sucrose made in 100 mM PBS, pH 7.4 and 0.02% sodium azide, Sigma) and cryo-sectioned using a cryo-stat (HN525 NX, Microm, Epredia) at the thickness of 30 µm (coronal). To determine the impact of chronic chemotherapy and RZ treatments on the function of the neurogenic niche, serial coronal brain sections (2-3 sections per brain, 8-10 brains per group) through the hippocampal formation were stained using free-floating

immunofluorescence protocols as described.[4] Doublecortin (DCX) staining was performed to label newly born, immature neurons as previously described.[5] Sections were PBS-washed, permeabilized by citrate, blocked using the normal donkey serum (NDS), and incubated overnight at 4°C with a rabbit anti-DCX primary antibody (1:200; Abcam, ab18723) and subsequently with a secondary antibody (donkey anti-Rabbit Alexa Fluor 568) for one hour at room temperature. Tissue sections were washed with PBS and counterstained using a nuclear stain (DAPI, 4′,6-diamidino-2-phenylindole; Invitrogen) and mounted on clean Superfrost Plus slides using Vectashield Plus anti-fade mounting medium (Vector Labs). DCX-positive cells were visualized using fluorescence microscopy as red. For the BrdU-NeuN dual-immunofluorescence staining, first, serial sections were rinsed in PBS (100 mM, pH 7.6, Gibco) and permeabilized to recover BrdU antigen via 50% formamide (made in 2× saline-sodium citrate (SSC) buffer; Sigma) at 68-70 °C for 2 hours followed by 2N HCl (at 37°C for 45 minutes). The sections were then treated with 0.1 M Borate buffer at pH 8.5 for 15 minutes at room temperature (20-25 °C). Sections were then washed in PBS, blocked by the serum (10% NDS made in PBS and 0.1% Triton, Sigma), and incubated overnight in primary antibodies, including rat anti-BrdU antibody (1:150; Abcam, ab6326) and rabbit anti-NeuN (1:500, Millipore) made in PBS, 0.1% Triton and 3% NDS. The sections were treated with donkey anti-rat Alexa Fluor 488 (1:150; Invitrogen) and anti-rabbit Alexa Fluor 568 (1:500, Invitrogen) for 1 hour. Next, the tissues were incubated in DAPI for 15 minutes. Immunostained sections were washed in PBS and mounted on the superfrost slides using Vectashield Plus anti-fade mounting medium (Vector Labs). BrdU-positive cells were visualized using fluorescence microscopy as green, and NeuN-positive cells as red. In silico analysis was conducted using Imaris (v9.2, BitPlane, Inc.) as described in the Methods.

For the IBA1-CD68 dual-immunofluorescence staining, first, serial sections were washed and permeabilized in PBS (100 mM, pH 7.6, Gibco) with 0.3% Tween-20 (3 washes, 5 min each) at room temperature. Subsequently, sections were treated with ice-cold 3% hydrogen peroxide (Sigma) and 10% methanol (Sigma) made in PBS for 30 minutes. The sections were then washed in PBS, blocked by 4% BSA (Sigma) made in PBS and 0.3% Tween-20, and incubated overnight in primary antibodies, including rabbit anti-IBA1 (1:500; Wako) and rat anti-mouse CD68 (1:500, AbD Serotec, Bio-Rad) made in PBS, 0.3% Tween-20 and 1% BSA. The next day, sections were washed in PBS and treated with goat anti-rabbit Alexa Fluor 488 (1:1000; Invitrogen) and goat anti-rat Alexa Fluor 647 (1:1000, Invitrogen) for 1 hour. Immunostained sections were washed in PBS and mounted on the superfrost slides using Vectashield Plus anti-fade mounting medium (Vector Labs). IBA1-positive cells were visualized using fluorescence microscopy as green, and CD69-positive cells as magenta (pseudo-color).

***In silico* volumetric quantification**

Immunostained sections were imaged with a laser-scanning confocal microscope (Nikon Eclipse Ti C2) equipped with a 40× oil-immersion objective lens (1.4 NA) and NIS element AR module (v4.3, Nikon). The high-resolution (1024 to 2048p) z stacks (0.5 µm thick) were scanned through the 25-30 µm thick section. An adaptive, 3D blinded deconvolution method (ClearView, Bitplane Inc., Zürich, Switzerland) was used to deconvolute images to improve the signal resolution with respective fluorescent wavelengths (510nm, green; 594 nm, red; 447 nm, UV; and 647 nm for IR range). The deconvoluted images were converted to IMS format for 3D algorithm-based Imaris analysis (v9.0, Bitplane Inc., Zürich, Switzerland). In Imaris, DCX, NeuN, BrdU, IBA1 and CD68 were 3D modeled using the surface-rendering tool to create the volume of individual neuronal and glial cells, BrdU, and CD68 expression. Using an unbiased, dedicated co-localization channel, the volume of BrdU labels or CD68 puncta on the surface of NeuN or IBA1 respectively were individually calculated by selecting each surface rendering at a distance of -0.5 µm to 0.5 µm. The total expression and the volume of each surface (immunoreactivity) or co-localized surfaces were used for quantitative comparison between the control and treatment groups. All *in silico* analyses were conducted using automated batch processing within the Imaris Arena module and criteria were applied uniformly for all experimental groups to avoid bias.

References

1. Lueptow LM. Novel Object Recognition Test for the Investigation of Learning and Memory in Mice. *J Vis Exp.* 2017(126).
2. Walf AA, Frye CA. The use of the elevated plus maze as an assay of anxiety-related behavior in rodents. *Nat Protoc.* 2007;2(2):322-328.
3. Curzon P, Rustay NR, Browman KE. Cued and Contextual Fear Conditioning for Rodents. In: JJ B, ed. *Methods of Behavior Analysis in Neuroscience.* Boca Raton (FL): CRC Press/Taylor & Francis; 2009.
4. Baulch JE, Acharya MM, Allen BD, et al. Cranial grafting of stem cell-derived microvesicles improves cognition and reduces neuropathology in the irradiated brain. *Proc Natl Acad Sci U S A.* 2016;113(17):4836-4841.
5. Christie LA, Acharya MM, Parihar VK, Nguyen A, Martirosian V, Limoli CL. Impaired cognitive function and hippocampal neurogenesis following cancer chemotherapy. *Clin Cancer Res.* 2012;18(7):1954-1965.
